# Hyperuricemia suppresses lumican, exacerbating adverse remodeling after myocardial infarction by promoting fibroblast phenotype transition

**DOI:** 10.1186/s12967-024-05778-4

**Published:** 2024-10-31

**Authors:** Zehao Zhuang, Ao Liu, Jinghong Zhang, Shuangjian Han, Lu Tang, Tingting Yu, Yiping Shi, Hui Li, Heng Yang, Peiyuan Bai, Yanhua Tang

**Affiliations:** 1 Department of Cardiovascular Surgery, The Second Affiliated Hospital of Nanchang University, Nanchang, Jiangxi Province China; 2 Department of Cardiology, Zhongshan Hospital, Shanghai Institute of Cardiovascular Diseases, Fudan University, Shanghai, China; 3 Institutes of Biomedical Sciences, Fudan University, Shanghai, China; 4 Department of Echocardiography, Zhongshan Hospital, Shanghai Institute of Cardiovascular Diseases, Shanghai Institute of Medical Imaging, Fudan University, Shanghai, China; 5 Department of Dermatology, Pudong New Area People’s Hospital, Shanghai, China; 6 Department of Cardiology, Shanghai Chest Hospital, Shanghai Jiao Tong University School of Medicine, Shanghai, China; 7 Department of Psychiatry, Shanghai Mental Health Center, Shanghai Jiao Tong University School of Medicine, Shanghai, China; 8 Department of Cardiology and Institute of Vascular Medicine, Peking University Third Hospital, Beijing, China

**Keywords:** Myocardial infarction, Hyperuricemia, Lumican, Fibroblasts, Cardiac remodeling

## Abstract

**Background:**

Hyperuricemia is independently associated with a poor prognosis in patients with myocardial infarction (MI). Furthermore, MI induces activation of the repair response in local fibroblasts, resulting in extracellular matrix accumulation that generates a stable fibrotic scar in the infarcted area. However, researchers have not determined whether hyperuricemia affects fibroblast activation and its involvement in postinfarction cardiac remodeling.

**Objectives:**

We aimed to trigger hyperuricemia by administering potassium oxonate in a mouse model of MI to evaluate the role of hyperuricemia in MI pathogenesis.

**Methods:**

Microarray datasets and single-cell sequencing data from gout patients, heart failure patients, and model mice were used to identify the underlying mechanisms responsible for the effect of hyperuricemia on MI progression. A hyperuricemia-related MI mouse model was established. Cardiac function was assessed, followed by sample collection and a uric acid assay. We conducted an enzyme-linked immunosorbent assay, histological detection, immunofluorescence, sequencing data processing, single-cell RNA-seq, and functional enrichment analysis. We then isolated and cultured cardiac fibroblasts and performed Western blotting, quantitative real-time polymerase chain reaction, and shRNA-mediated lumican knockdown assays.

**Results:**

Hyperuricemia decreased cardiac function, increased mortality, and aggravated adverse fibrosis remodeling in mice after MI. These outcomes were closely related to reduced levels of fibroblast-derived lumican. This reduction activated the TGF-β/SMAD signaling pathway to induce aberrant myofibroblast activation and extracellular matrix deposition in the infarcted area. Furthermore, lumican supplementation or uric acid-lowering therapy with allopurinol alleviated hyperuricemia-mediated abnormal cardiac remodeling.

**Conclusion:**

Hyperuricemia aggravates postinfarction cardiac remodeling by reducing lumican expression and promoting fibroblast phenotype transition. We highlight the clinical importance of lowering uric acid levels in hyperuricemia-related MI to prevent adverse ventricular remodeling.

**Supplementary Information:**

The online version contains supplementary material available at 10.1186/s12967-024-05778-4.

## Introduction

Myocardial infarction (MI) is a serious cardiovascular disease characterized by sudden onset, severe disease, and poor prognosis [1] and is one of the leading causes of death and disability worldwide, placing a tremendous burden on patients, families, and societies [2]. The widespread application of interventional thrombolytic techniques and the development of novel drugs have reduced MI-related mortality, yet the mortality and adverse prognoses associated with heart failure after MI remain persistently high, a situation that cannot be ignored [3–5]. Therefore, novel and complementary therapies are essential to ensure timely treatment that limits the extent of myocardial injury and prevents subsequent complications.

Metabolic disorders induced by an unhealthy lifestyle (including obesity, dyslipidemia, and insulin resistance) are responsible for increased MI incidence [6, 7]. Furthermore, hyperuricemia is defined as an elevated serum uric acid level and is recognized as a precursor to the development of metabolic and cardiovascular diseases [8, 9]. In humans, hyperuricemia is defined as a serum uric acid level of ≥ 7.0 mg/dL (416.0 µmol/L) in men or ≥ 6.0 mg/dL (357.0 µmol/L) in women. In a mouse model, hyperuricemia was induced by the oral administration of potassium oxonate (Po) at 250 mg/kg per day, which resulted in a 1.5–2.1-fold increase in the serum urate concentration within 7 days [10]. A population-based study revealed that hyperuricemia is independently associated with poor prognosis in patients with MI, suggesting that assessing plasma uric acid levels may be useful for MI risk stratification [11]. Moreover, lowering uric acid levels with allopurinol reduces MI risk [12]. Currently, preventive and therapeutic measures for the adverse outcomes of hyperuricemia-induced MI are limited. Therefore, there is an urgent need to explore the potential mechanisms associated with hyperuricemia and its adverse prognosis in these patients.

Collagen-based scar repair following acute MI facilitates the formation of organized fibrotic tissue to replace necrotic areas and mitigate the risk of cardiac rupture [13, 14]. This process is driven by the activation of cardiac fibroblasts, which transform into myofibroblasts in response to injury or stress [15]. These activated fibroblasts release the profibrotic cytokine transforming growth factor β1 (TGF-β) and initiate the TGF-β/SMAD signaling pathway post-MI [16–18]. Although myofibroblast differentiation is a physiological response to acute MI [19], its persistence contributes to maladaptive remodeling, functional decline, and heart disease progression [15, 20]. However, little is known regarding whether hyperuricemia influences myofibroblast differentiation and function during MI progression.

We aimed to induce hyperuricemia in a mouse model of MI through Po administration to investigate the role of hyperuricemia in the pathogenesis of MI. Our study focused on evaluating the effects of hyperuricemia on cardiac fibroblasts and the TGF-β/SMAD signaling pathway and explored the potential role of lumican as a biomarker.

## Materials and methods

### Animal model

C57BL/6 male mice (6‒8 weeks old, weighing 20–25 g) were procured from the Model Organisms Center, Inc. (Shanghai, China). To induce hyperuricemia-related MI, the mice received intraperitoneal injections of Po (CAS 2207-75-2, TargetMol, MA) at a dosage of 300 mg/kg dissolved in 0.5% sodium carboxymethyl cellulose for 21 consecutive days. On the seventh day, the Po-treated mice underwent permanent ligation of the left anterior descending (LAD) artery following established protocols [21]. Permanent LAD ligation or a sham operation was performed on experimental mice as previously described. The mice were anesthetized with 2% isoflurane, a 1.2 cm incision was made on the left chest, and a 6–0 silk suture was used to ligate the LAD artery 2–3 mm below the left auricle. Mice that did not survive the first 24 h were excluded. Sham-operated mice underwent the same procedure without ligation. At specified time points, the mice were euthanized, and the tissues were collected for analysis.

The control group received an equal volume of 0.5% sodium carboxymethyl cellulose and underwent sham surgery without LAD artery ligation. Mice that did not survive beyond 24 h postsurgery were excluded. Uric acid levels were reduced in vivo by administering 125 mg/L allopurinol (CSN16291, CSNpharm, Chicago, IL) dissolved in the drinking water for 14 days. All procedures adhered to the guidelines of the Institutional Animal Care and Use Committee of Nanchang University.

## Echocardiography

On the 14th day post-MI surgery, cardiac function was assessed using a Vevo 2100 high-frequency high-resolution ultrasound system (Fujifilm VisualSonics, Inc., Toronto, Canada). The mice were anesthetized with isoflurane and gently secured in the supine position on a heated operating table maintained at a constant temperature of 37 °C. To ensure optimal imaging conditions while preserving the consciousness of the mice, a carefully titrated lower dose of isoflurane was administered throughout the procedure.

Using the advanced biplane area-length method, the end-diastolic volume (EDV) and end-systolic volume (ESV) were precisely determined, paving the way for accurate calculation of the left ventricular ejection fraction (LVEF) through the formula LVEF (%) = [(EDV‒ESV)/EDV] × 100%. Two-dimensional M-mode images were obtained in both the long-axis and short-axis orientations to measure the left ventricular end-diastolic internal diameter (LVEDD) and left ventricular end-systolic internal diameter (LVESD). Subsequently, the formula FS (%) = ([LVEDD-LVESD])/LVEDD × 100% was used to calculate short-axis shortening (FS) rate. To ensure objectivity, images were analyzed by an operator who was blinded to the treatment groups.

## Sample collection and uric acid assay

The mice were subjected to a 15-h fasting period before postmortem examination. Following isoflurane anesthesia, blood samples were collected from the inner canthus. A sterile capillary tube was inserted into the engorged venous plexus to collect 0.2–0.3 mL of blood. Bleeding was controlled with a sterile cotton ball after sample collection. This method allowed for repeated sampling with minimal distress. The samples were collected in clean ethylenediaminetetraacetic acid (EDTA) bottles and allowed to clot at 20–25 °C for approximately 1 h, followed by centrifugation at 10,000 × g for 15 min to obtain the serum. Serum samples were stored at -20 °C until use. Serum uric acid levels were determined via a uric acid (UA) enzymatic colorimetric assay (ab65344; Abcam, Cambridge, UK) following the manufacturer’s instructions.

## Enzyme-linked immunosorbent assay (ELISA)

Blood samples were collected in heparin-coated tubes after euthanasia and centrifuged at 1000 × g for 15 min at 4 °C to separate the plasma, which was stored at -80 °C. Plasma c-TNT (SEKM-0150, Solarbio, China, Beijing), CK-MB (ab285231, Abcam), lumican (RK08235, ABclonal, China Wuhan), and active TGF-β1 (MB100B, R&D Systems, Minneapolis, MN, USA) levels were measured with the appropriate ELISA kits according to the manufacturers’ instructions.

## Histological analyses

After blood residues were removed with a phosphate-buffered solution, the heart tissues were fixed in a 4% formalin solution and embedded in paraffin. Histological staining of the scar tissue at the papillary muscle level was performed, and the tissues were sliced into 5 μm-thick sections. To assess infarct size and wall thickness, tissue sections were stained with hematoxylin and eosin. Masson’s trichrome was used to determine the collagen density in the fractional area as previously described [22]. Collagen was highlighted with aniline blue, which stains collagen fibers blue, in contrast with the red-stained muscle fibers. Quantification was performed using ImageJ software (NIH, MD).

### Immunofluorescence analyses

For immunofluorescence, cardiac tissues were embedded in optimal cutting temperature compound. Cryosections (5 μm) were fixed in a 4% paraformaldehyde solution for 15 min at 4 °C. After being blocked with 5% bovine serum albumin, the tissues were incubated with the primary antibody overnight at 4 °C. Alexa fluorescence-conjugated secondary antibodies were used to visualize the signals. The following antibodies were used: anti-Lumican (A11593, ABclonal, 1:200, Wuhan, China), anti-α-SMA (A5228, Sigma‒Aldrich, 1:200, St. Louis, MO), anti-collagen 1 (14695-1-AP, Proteintech, Wuhan, China), anti-fibronectin (A12932, ABclonal, 1:200), anti-CD68 (ab955, Abcam), anti-Ly6G (127602, Biolegend, San Diego, CA), anti-CD31 (561810, BD Biosciences, San Jose, CA), anti-c-TnT (A11826, Invitrogen, Carlsbad, CA), 488 nm Alexa fluorescence-conjugated secondary antibodies (A-11001, Thermo Fisher Scientific, Waltham, MA), and 555 nm Alexa fluorescence-conjugated secondary antibodies (A-21428, Thermo Fisher Scientific). According to the manufacturer’s instructions, terminal deoxynucleotidyl transferase dUTP nick end labeling (TUNEL) staining was performed using an In Situ Apoptosis Detection Kit (11684817910, Roche, Basel, Switzerland). To determine the cross-sectional area of the cardiomyocytes, the sections were counterstained with Alexa Fluor 488-conjugated wheat germ agglutinin (WGA) (W11261, Thermo Fisher Scientific). The stained sections were examined via a state-of-the-art laser-scanning confocal microscope (Carl Zeiss, Oberkochen, Germany).

## Sequence data processing

Two microarray datasets (GSE160170 and GSE5406) and one single-cell RNA sequencing dataset (E-MTAB-7895) were selected from the Gene Expression Omnibus database (https://www.ncbi.nlm.nih.gov/geo) and ArrayExpress (https://www.ebi.ac.uk/biostudies/arrayexpress/). The GSE160170 dataset comprises gene expression profiles of long noncoding RNAs (lncRNAs) and mRNAs in the peripheral blood mononuclear cells of patients with primary gout and healthy control individuals. The Acuity software (Molecular Devices) performed LOWESS nonlinear normalization on the data. Genes detected in at least 50% of the samples were included, and a total of 3,409 genes were ultimately analyzed. Differentially expressed genes (DEGs) were identified via the Wilcoxon test. Genes with a log_2_FC (fold change, FC) > 0.585 and a P value < 0.05 were considered significant DEGs. The GSE5406 dataset featured the gene expression profile of the left ventricular myocardium at the time of cardiac transplantation from patients with advanced idiopathic or ischemic cardiomyopathy, with that from an unused donor heart at the time of harvest serving as a nonfailing control. LOWESS nonlinear normalization was performed on the data via Acuity software (Molecular Devices). Genes detected in at least 50% of the samples were included, and 386 genes were analyzed. DEGs were identified via the Wilcoxon test. Genes with log2FC > 0.585 and a P value < 0.05 were considered significant DEGs. The E-MTAB-7895 dataset featured single-cell gene expression data of cardiac interstitial cells at homeostasis and at 1, 3, 5, and 14 days after acute MI and was processed via the Cell RangerTM v3.0.1 pipeline (http://10xgenomics.com).

## Single-cell RNA-seq analysis

The analysis of single-cell samples obtained from control and MI mice involved several steps. Initially, data integration was performed by combining samples from both groups. With the use of the Seurat package (v4.0.1) in R, standard procedures were followed, including filtration, identification of genes exhibiting high variability, reduction in dimensionality, and clustering of cells. Cells that were deemed low-quality, expressed fewer than 200 genes or expressed in fewer than three cells were excluded from further analysis. Additionally, cells expressing more than 2,500 genes were eliminated to mitigate potential doublet occurrences. The cells whose mitochondrial gene expression exceeded 5% were also filtered out because of poor quality. Normalization was carried out by scaling the gene expression levels of the remaining cells, followed by logarithmic transformation of the results, with a multiplication factor of 10,000. Highly variable genes were identified through standard deviation measures and subjected to subsequent analyses.

Data scaling involves a linear transformation process, a standard preprocessing step aimed at adjusting the mean expression to zero and the variance to one across cells, in preparation for dimensionality reduction techniques. Principal component analysis was performed via a graph-based clustering approach, with the resolution parameter set to 0.5. The analysis was conducted on the top 2,000 most variable genes to reduce dimensionality. Thirty principal components were chosen on the basis of their significance. The Louvain algorithm was employed to classify cells into distinct clusters. To visualize the resulting clusters in a two-dimensional space, uniform manifold approximation and projection (UMAP) was utilized. Identification of DEGs within each cluster was conducted via the Wilcoxon test. DEGs with a log2-fold change > 0.25 and an adjusted p value (pvals_adj) < 0.05 were considered significant.

### Functional enrichment analysis

Gene ontology (GO) biological process (bp) enrichment and pathway analyses, including Kyoto Encyclopedia of Genes and Genomes (KEGG), reactome gene set, and canonical pathway analyses, were performed on the genes of interest via Metascape. Enriched GObp terms and pathways associated with *p* < 0.05 were considered significant.

Primary fibroblasts from neonatal rat cardiac fibroblasts were isolated and cultured from 24–48-h-old Sprague‒Dawley rats after birth. First, the heart was excised after the surrounding connective tissue was removed. The tissue was subsequently incubated for 15 min at 37 °C in a digestion mixture containing 0.05% trypsin-EDTA and 0.1 mg/mL DNase 1. This step was repeated 8–10 times. After each digestion, the supernatants were collected, mixed with an equal amount of complete Dulbecco’s modified Eagle’s medium (DMEM), and centrifuged at 1,000 rpm for 5 min. After centrifugation, the pellet containing cardiac fibroblasts was resuspended in complete DMEM and plated onto dishes. After 90 min, the culture medium was changed to remove nonadherent cells. The primary rat cardiac fibroblast cultures were used upon reaching 80% confluence. To explore the effect of hyperuricemia on rat cardiac fibroblasts, the cells were treated with UA (100 mg/L) for 24 h and collected for assays. To evaluate the impact of lumican supplementation on the activation and fibrosis of rat cardiac fibroblasts, the cells were treated with 10 ng/mL TGFβ1 (100–21, PeproTech) and 100 nM recombinant lumican (RP00216, ABclonal) for 24 h.

### Western blotting

Heart tissues from the infarcted area and rat cardiac fibroblasts were lysed via radioimmunoprecipitation assay lysis buffer (P0013B, Beyotime, Shanghai, China) and quantified via a bicinchoninic acid protein assay kit (P0009, Beyotime) according to the manufacturer’s instructions. Equal amounts of boiled protein extracts were separated via 10% SDS‒PAGE gels (PG112, Epizyme, Shanghai, China) and transferred to methanol-preactivated polyvinylidene difluoride membranes. The membranes were incubated with 5% skim milk for 1 h and then with primary antibodies overnight at 4 °C. Next, the membranes were incubated with a horseradish peroxidase-linked secondary antibody for 1 h at room temperature. The protein bands were visualized using an enhanced chemiluminescence reagent (SB-WB004, Sharebio, Shanghai, China) and quantified via ImageJ software. The primary antibodies used were as follows: anti-Lumican (A11593, ABclonal, 1:1,000), anti-p-Smad2 (8828 S, Cell Signaling Technology, Danvers, MA, USA), anti-Smad2 (A19114, ABclonal, 1:1,000), anti-p-Smad3 (AP0554, ABclonal, 1:1,000), anti-Smad3 (A19115, ABclonal, 1:1,000), anti-Smad4 (A5657, ABclonal, 1:1,000), anti-α-SMA (A5228, Sigma‒Aldrich, 1:1,000), anti-collagen 1 (14695-1-AP, Proteintech, 1:1,000), anti-fibronectin (A12932, ABclonal, 1:1,000), and anti-β-actin (AC026, ABclonal, 1:50,000).

### Quantitative real-time polymerase chain reaction (PCR)

Total RNA samples from the infarcted area and cardiac fibroblasts were prepared using the Trizol reagent (Invitrogen), according to the manufacturer’s protocol. Total RNA (1 µg) was converted to cDNA using the PrimeScript RT Master Mix (Takara Bio Inc., Kusatsu) as recommended by the manufacturer. The resulting cDNA was amplified for 40 cycles by real-time PCR, and detection was achieved using SYBR Green Mix (Applied Biosystems, Waltham, MA, USA). Each sample was analyzed in triplicate and normalized to a reference RNA. Relative expression levels were quantitated using the ΔΔCt method. The sequences of the PCR primer are shown in Additional File 1.

### shRNA-mediated lumican knockdown assay

To achieve gene knockdown, we acquired a specific shRNA targeting the gene of interest from Obio Technology Corporation and a lentiviral packaging service. The shRNA was cloned and inserted into a lentiviral vector, and high-titer lentiviral particles were generated for efficient transduction into target cells. This process allows for stable integration and sustained knockdown of the target gene, enabling further investigation of its functional role. All experiments were conducted in compliance with biosafety guidelines. To assess the impact of lumican knockdown on the fibrosis process in cardiac fibroblasts, 10 ng/mL TGFβ1 was added to the cell culture medium for 24 h to activate the fibroblasts.

### Statistical analysis

The data were analyzed via GraphPad Prism version 8 (GraphPad Software, San Diego, CA, USA). All data are presented as the means ± standard errors. Unpaired Student’s t tests or nonparametric Mann‒Whitney U tests were used to compare means between groups. For multiple comparisons, one-way analysis of variance followed by Bonferroni’s post hoc analysis or a nonparametric Kruskal‒Wallis test with Dunn’s multiple comparisons test was performed. Kaplan–Meier survival curves were compared via the log-rank test. A value of *P* < 0.05 was considered significant.

## Results

### Hyperuricemia aggravates adverse cardiac remodeling after MI

**Fig. 1:**
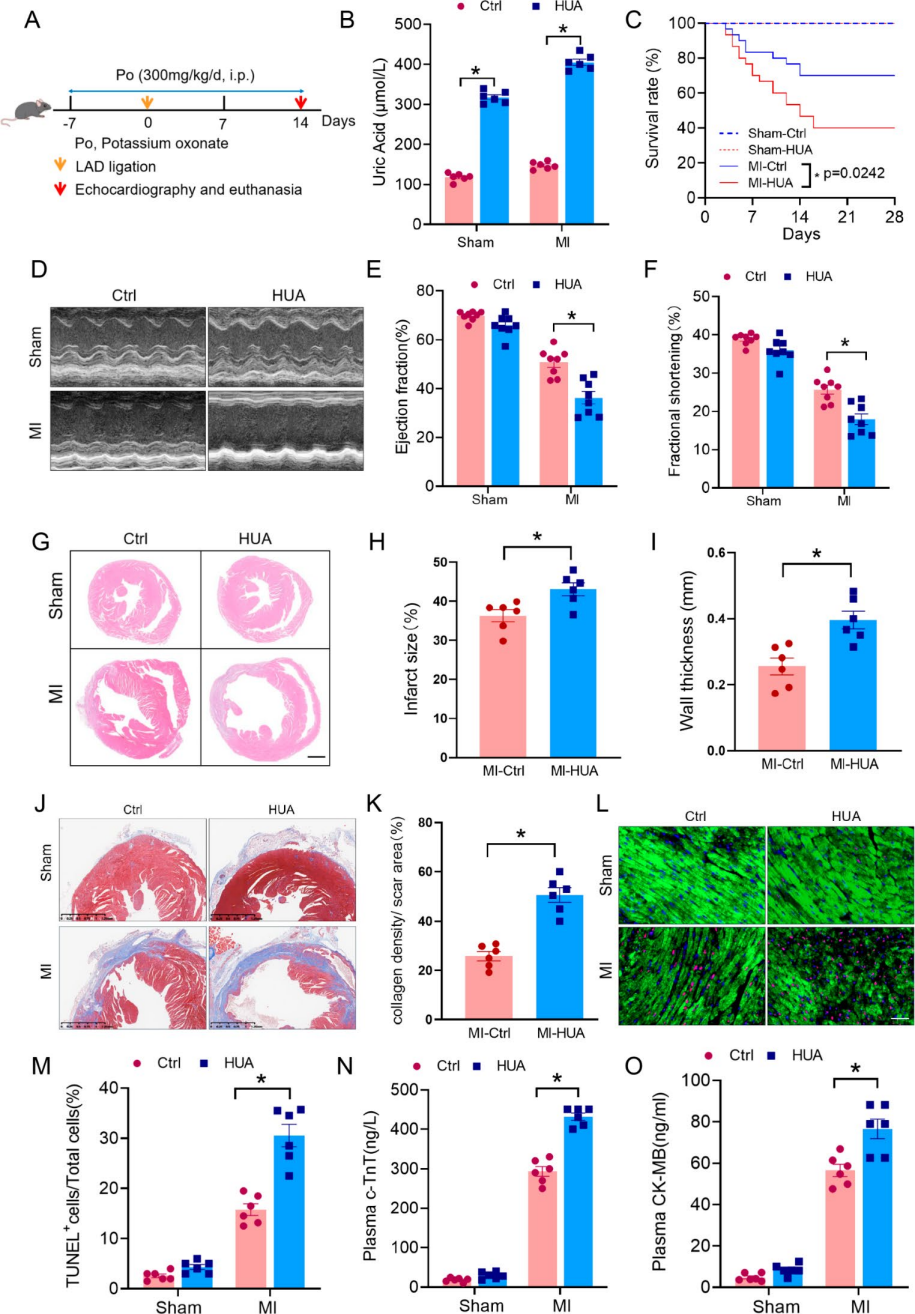
Hyperuricemia aggravates adverse cardiac remodeling after MI. **(A)** Schedule for establishing hyperuricemia and MI in a mouse model. **(B)** Serum levels of uric acid (UA) were measured in potassium oxonate-treated mice subjected to MI or sham surgery on day 14 (*n* = 6). **(C)** Kaplan–Meier survival curves for each group of mice. **(D)** Representative M-mode images of heart tissue from mice obtained on day 14. **(E, F)** Quantification of the ejection fraction and fractional shortening measured via echocardiography on day 14 (*n* = 8). **(G)** Representative H&E images of cardiac tissues obtained from hyperuricemic mice on day 14 after MI or sham surgery. **(H, I)** Quantitative analysis of infarct size and wall thickness (*n* = 6). **(J)** Representative images of Masson’s trichrome-stained cardiac tissues obtained from hyperuricemic mice after MI or sham surgery on day 14. **(K)** Areas positive for Masson’s trichrome staining were quantified to determine the collagen density in the hearts of the mice in the indicated groups (*n* = 6). **(L, M)** Representative immunofluorescence images and quantitative analysis of TUNEL and nuclear 4’-6-diamidino-2-phenylindole (DAPI) in c-TnT-positive cardiomyocytes obtained from hyperuricemic mice with MI or sham surgery on day 14 (*n* = 6). **(N, O)** c-TnT and CK-MB expression levels in mouse serum were assessed by ELISA (*n* = 6). The data are expressed as the means ± standard errors. Statistical analysis was performed via Student’s t test **(B, E, G, I, K, L, and M)** and the log-rank test **(C)**. *p < 0.05. MI, myocardial infarction

To establish the hyperuricemia-combined MI model, the mice were intraperitoneally administered Po (300 mg/kg) daily for 7 days, followed by LAD artery ligation surgery. The experiment was concluded with sample collection 14 days after MI surgery (Fig. 1A). Here, we confirmed serum UA levels in mice using a UA enzymatic colorimetric assay. The results revealed that Po significantly elevated the UA content in mouse serum compared with that in the control group, suggesting the successful establishment of hyperuricemia (Fig. 1B).

We explored the effects of hyperuricemia on post-MI survival. The survival rate of hyperuricemia-related MI mice was significantly decreased (Fig. 1C). To further evaluate the effect of hyperuricemia on cardiac function after MI, we assessed echocardiographic parameters, including LVEF and FS. We found that hyperuricemia significantly impaired cardiac function in MI mice compared with MI mice without PO (Fig. 1D–F). Compared with the control, hyperuricemia aggravated ventricular remodeling 14 days after MI, as illustrated by an increased infarct area and wall thickness in the infarcted area (Fig. 1G–I). The collagen density in the infarcted area was also significantly increased (Fig. 1J, K).

The number of TUNEL-positive cardiomyocytes in the heart border area was significantly greater in hyperuricemia-related MI mice than in control mice on day 14 (Fig. 1L, M), suggesting increased apoptosis. Moreover, elevated plasma levels of c-TnT and CK-MB (standard markers of myocardial injury) suggested enhanced myocardial injury (Fig. 1N, O). Collectively, these results suggest that hyperuricemia accelerates myocardial dysfunction and exacerbates adverse cardiac remodeling after MI.

### Reduced lumican expression in hyperuricemia-related MI

**Fig. 2:**
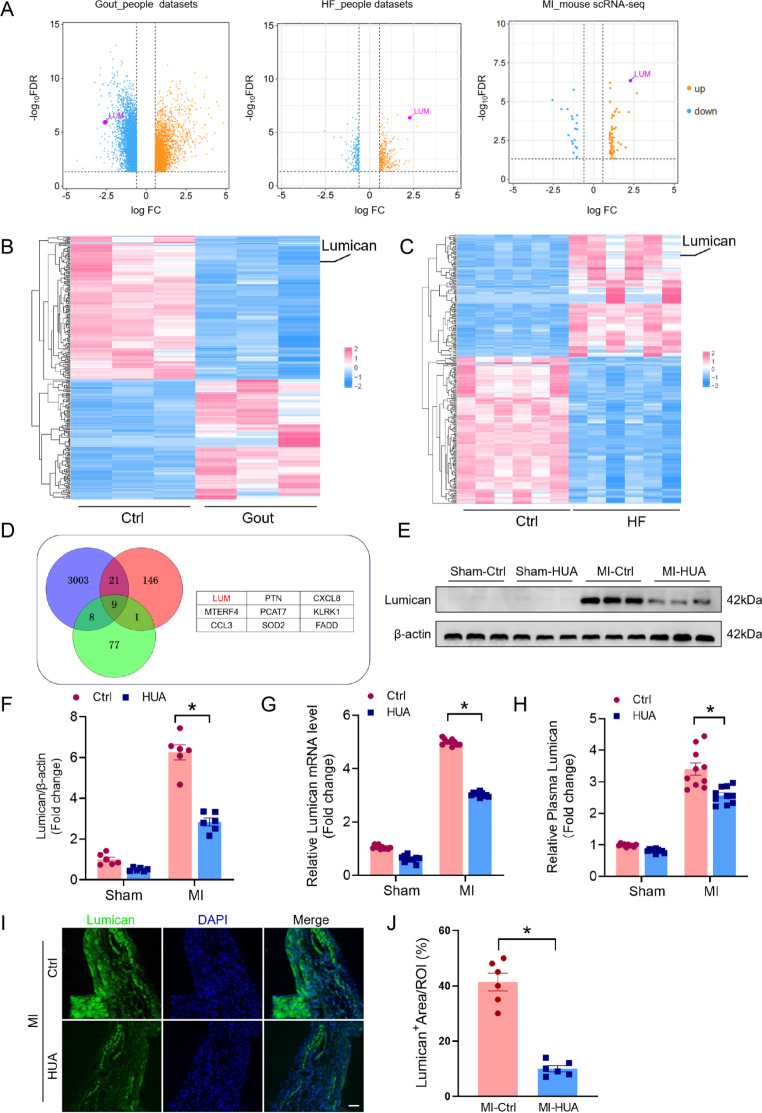
Decreased lumican levels are associated with enhanced cardiac remodeling in hyperuricemia-related MI. **(A)** Volcano plots showing differentially expressed genes in patients with gout, patients with heart failure, and MI mice compared with controls through microarray datasets or single-cell RNA sequencing. **(B, C)** Heatmaps depicting DEGs between patients with gout and healthy individuals **(B**) and between patients with heart failure and healthy individuals **(C)**. **(D)** Venn analysis of DEGs from patients with gout, heart failure, and MI mice compared with controls. **(E)** Lumican protein levels in the infarct area of hyperuricemic mice after MI surgery determined by western blotting on day 14. **(F)** Quantitative analysis of lumican expression (*n* = 6). **(G)** Lumican mRNA expression in the infarct area of hyperuricemic mice after MI surgery was assessed by qPCR on day 14 (*n* = 6). **(H)** Plasma levels of lumican were evaluated by ELISA (*n* = 10). **(I)** Immunofluorescence staining for lumican in the infarct area of hyperuricemic mice on day 14 after MI surgery (*n* = 6). The data are expressed as the means ± standard errors. Statistical analysis was performed via Student’s t test. *p < 0.05. DEG, differentially expressed gene; MI, myocardial infarction

Next, we sought to understand how hyperuricemia affects MI progression. Gout is a painful form of arthritis that occurs when high levels of UA in the blood cause the formation and accumulation of crystals around the joints, where heart failure is a frequent complication of MI [23, 24]. To this end, we analyzed microarray datasets from patients with gout (GSE160170) or heart failure (GSE5406) and single-cell RNA-sequencing data (E-MTAB-7895) from MI mice (Fig. 2A-C). We observed a significant negative correlation between lumican levels and hyperuricemia (Fig. 2A, B). Additionally, lumican expression was markedly elevated in patients with heart failure and in MI mice (Fig. 2A, C). Hence, we hypothesized that lumican might play a role in hyperuricemia-induced cardiac injury and that its increased expression following cardiac damage could serve as a compensatory mechanism. In total, 3,003, 146, and 77 genes were identified as being differentially expressed in gout, heart failure, and MI mice, respectively, compared with their respective control groups. Nine genes appeared at the intersection of the DEGs (Fig. 2D). Among these genes, the relationship between lumican and the extracellular matrix (ECM) of fibroblasts is the most direct, as it is directly involved in the assembly and maintenance of the ECM. We further employed western blotting and qPCR to evaluate lumican expression levels in hyperuricemia-related MI mice. In MI mice, lumican mRNA and protein expression significantly increased; however, hyperuricemia suppressed this increase (Fig. 2E-G). Compared with the control conditions, hyperuricemia also decreased the plasma levels of lumican (Fig. 2H). Moreover, immunofluorescence staining revealed that the expression of lumican in the infarcted cardiac tissue decreased in response to hyperuricemia after MI compared with that in controls (Fig. 2I, J).

### Reduced lumican levels accelerate adverse cardiac remodeling via the TGF-β signaling pathway

**Fig. 3:**
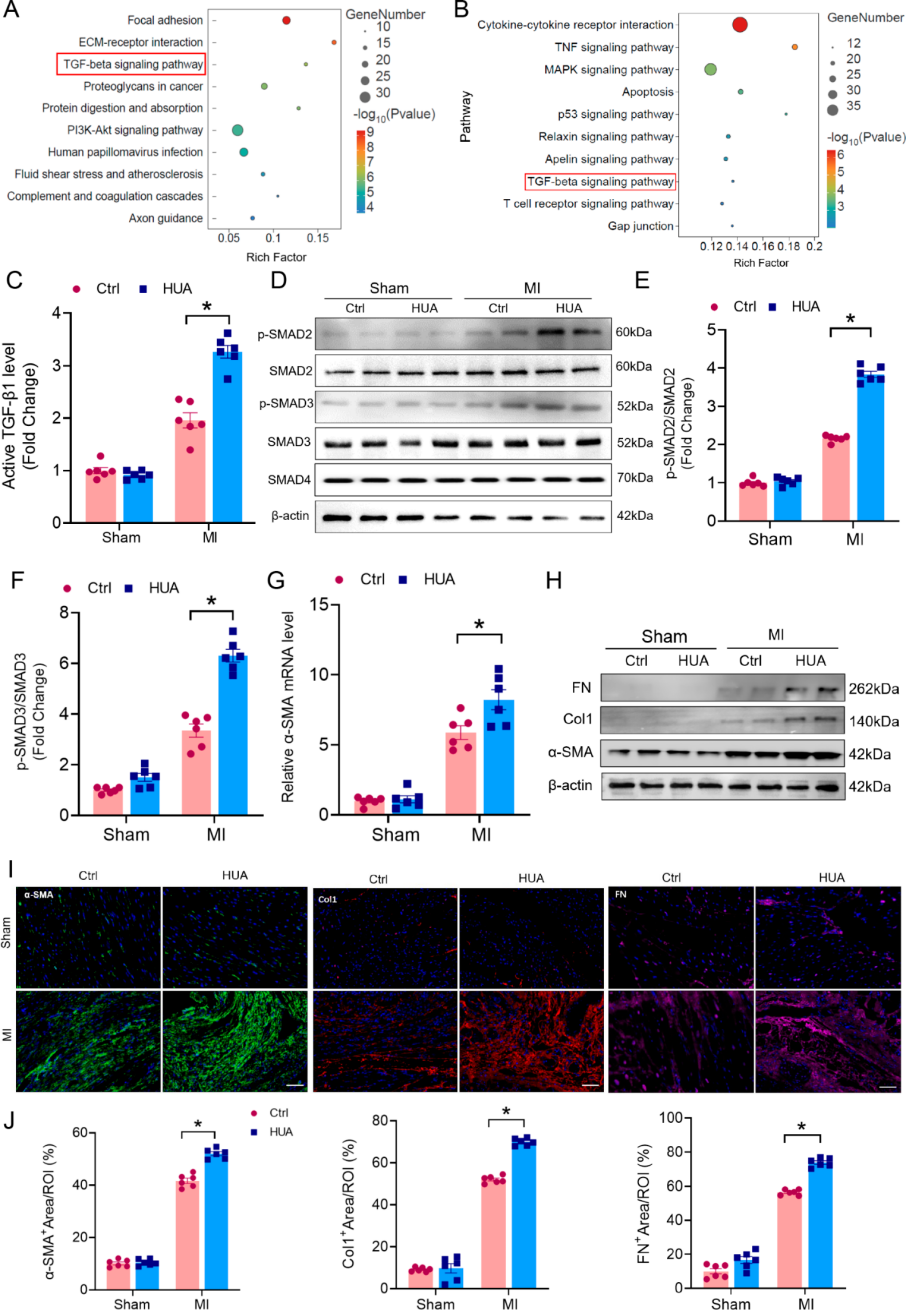
Lumican accelerates adverse cardiac remodeling by inhibiting TGFβ/Smad signaling. **(A, B)** KEGG enrichment analysis of DEGs between patients with heart failure and healthy individuals (A) and between patients with gout and healthy individuals **(B)**, **(C)** Serum levels of active TGF-β1 detected by ELISA in hyperuricemic mice after MI surgery (*n* = 6). **(D)** Western blot analysis of p-Smad2, total Smad2, p-Smad3, total Smad3, and Smad4 in the infarcted area of hyperuricemic mice after MI surgery. **(E, F)** Quantitative analysis of p-SMAD2 and p-SMAD3 expression (*n* = 6). **(G)** α-SMA mRNA expression in the infarcted area of hyperuricemic mice after MI surgery was assessed via qPCR. **(H)** Western blot analysis of α-SMA, collagen 1, and fibronectin in the infarct area of hyperuricemic mice after MI surgery. **(I, J)** Immunofluorescence staining and quantification of α-SMA, collagen 1, and fibronectin levels in the infarct areas of hyperuricemic mice after MI surgery. The data are expressed as the means ± standard errors. Statistical analysis was performed via Student’s t test. *p < 0.05. DEG, differentially expressed gene; KEGG, Kyoto Encyclopedia of Genes and Genomes; MI, myocardial infarction

Next, we analyzed microarray datasets to identify transcriptomic changes caused by lumican dysregulation in patients with heart failure and in those with gout. The KEGG analysis of the DEGs in these patients revealed that the TGF-β signaling pathway was activated in patients with heart failure and gout (Fig. 3A, B). As a member of the small leucine-rich proteoglycan family, lumican controls cell proliferation or fibroblast activation during fibrosis progression, suggesting that lumican is a potential endogenous modulator of the TGF-β signaling pathway [25–27].

The decreased lumican levels prompted us to determine whether the TGF-β signaling pathway was activated in hyperuricemia-related MI. We quantified active TGF-β1 levels in cardiac tissues via ELISA. Compared with those in control mice, active TGF-β1 levels were significantly increased in hyperuricemia-related MI mice (Fig. 3C). Moreover, the expression of molecules downstream of the TGF-β signaling pathway, p-SMAD2/3, as determined by western blotting, was increased in hyperuricemia-related MI mice (Fig. 3D-F).

Although effective TGF-β signaling pathway-mediated ECM formation in the infarct area is necessary to prevent heart rupture, excessive deposition of the ECM in the myocardium may distort its architecture, facilitate the progression of arrhythmia and cardiac dysfunction, and influence the clinical course and outcome in patients with heart failure [28, 29]. The levels of the ECM components α-SMA, collagen 1, and fibronectin were significantly greater in the infarcted area of hyperuricemia-related mice than in those of control mice after MI (Fig. 3G-J, Supplementary Fig. S1). These results suggest that reduced lumican may accelerate adverse cardiac remodeling by activating TGFβ/SMAD signaling in hyperuricemia-related MI.

### Lumican is derived from cardiac myofibroblasts in hyperuricemia-related MI

**Fig. 4:**
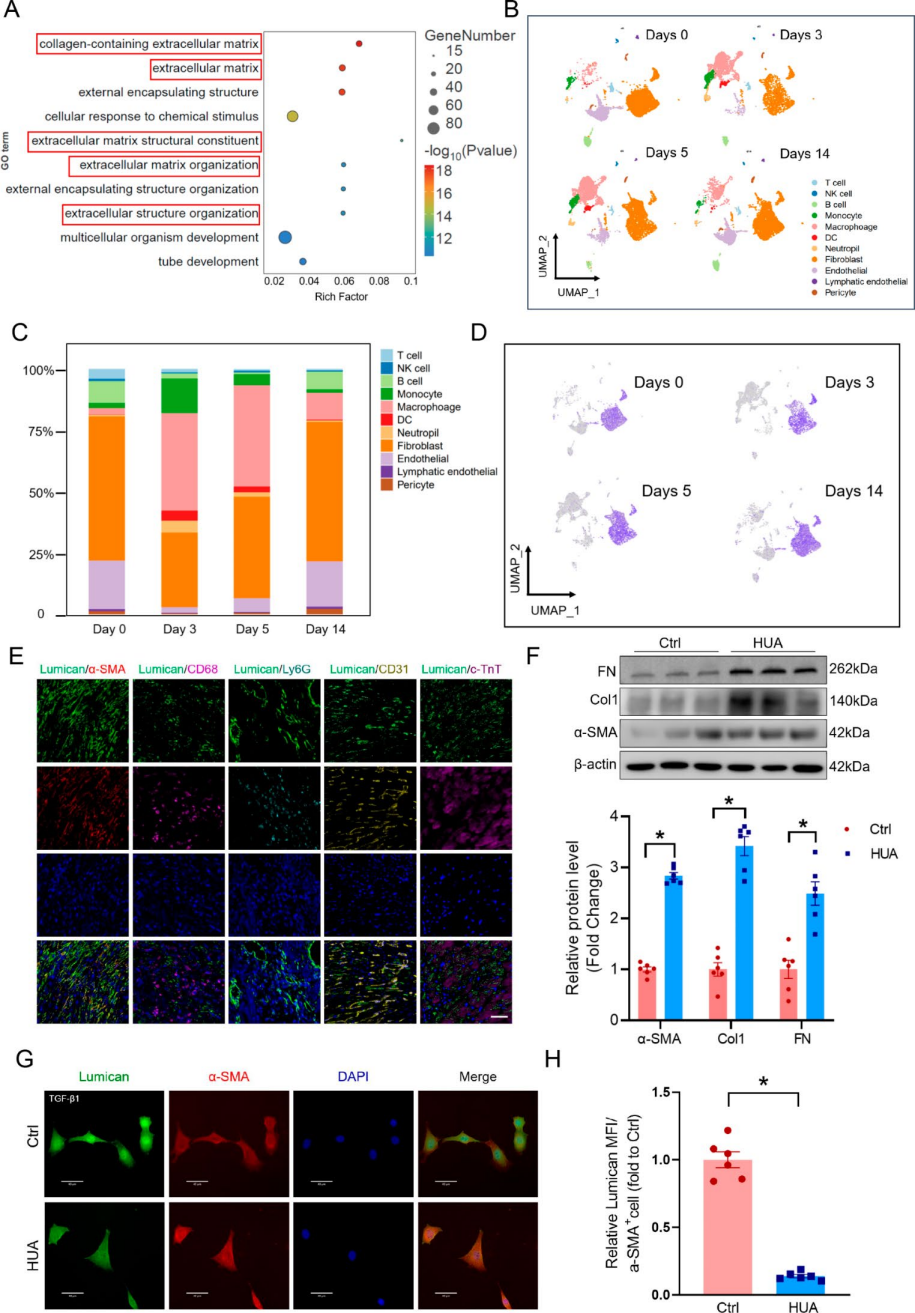
Lumican is derived from cardiac myofibroblasts. **(A)** GO enrichment analysis of DEGs between MI and control mice. **(B)** UMAP of 11 main cell types in the infarct area after MI surgery at different time intervals. **(C)** Percentages of different cell types identified in the infarct area tissues. **(D)** UMAP of lumican expression differences in different cell types identified in the infarct area tissues. **(E)** Representative images of lumican (green), α-SMA (red), CD68 (purple), Ly6G (blue), CD31 (yellow), and c-TnT (dark blue) immunostaining in hyperuricemic murine hearts after MI surgery on day 14. **(F)** α-SMA, collagen 1, and fibronectin expression in uric acid-treated cells evaluated by western blotting in rat primary cardiac fibroblasts treated with uric acid (100 mg/L) for 24 h. **(G, H)** Representative immunostaining image and quantitative analyses of lumican (green) and α-SMA (red) in uric acid-treated rat primary cardiac fibroblasts. The data are expressed as the means ± standard errors. Statistical analysis was performed via Student’s t test. *p < 0.05. DEG, differentially expressed genes; GO, Gene Ontology; MI, myocardial infarction; UMAP, uniform manifold approximation and projection for dimension reduction

We then determined which cell type in the infarcted area was responsible for abnormal lumican expression. GO enrichment analysis of DEGs between MI and control mice revealed that genes related to the ECM were enriched (Fig. 4A). To further elucidate the cellular dynamics and microenvironment composition during MI progression, single-cell RNA sequencing data from the cardiac infarct areas of MI mice on days 0, 3, 5, and 14 were collected for analysis. Eleven distinct cell populations, including T cells, natural killer cells, B cells, monocytes, macrophages, dendritic cells, neutrophils, fibroblasts, endothelial cells, lymphatic epithelial cells, and pericytes, were identified via UMAP (Fig. 4B). The cell number and proportion of cellular clusters varied significantly during MI progression (Fig. 4C, Supplementary Fig. S2). Compared with those on days 3 and 5, the number of fibroblasts on day 14 differed and was greater (Fig. 4C). On days 3, 5, and 14, the number of fibroblasts gradually increased, indicating their central role in hyperuricemia-related MI progression (Supplementary Fig. S2).

We then evaluated the differences in lumican expression in different cell types in the infarcted tissue. The UMAP results revealed that lumican was derived mainly from fibroblasts (Fig. 4D). To further explore the origin of lumican in the infarcted area in hyperuricemia-related MI, relative lumican expression was characterized in fibroblasts (marked by α-SMA), macrophages (marked by CD68), neutrophils (marked by Ly6G), endothelial cells (marked by CD31), and cardiomyocytes (marked by c-TnT). We found that in hyperuricemia-related MI, lumican was derived mainly from fibroblasts in the infarct area rather than from macrophages, neutrophils, endothelial cells, or cardiomyocytes (Fig. 4E). The protein expression levels of α-SMA, collagen 1, and fibronectin in primary rat cardiac fibroblasts were significantly increased after UA treatment (100 mg/L for 24 h) to mimic the hyperuricemia microenvironment (Fig. 4F). Immunofluorescence staining confirmed increased expression of α-SMA and collagen 1 in primary cardiac fibroblasts after UA treatment (Fig. 4G–H, Supplementary Fig. S3H). Collectively, these results show that lumican is derived mainly from cardiac myofibroblasts in hyperuricemia-related MI.

### Disruption of lumican expression promotes fibroblast phenotype transition

**Fig. 5:**
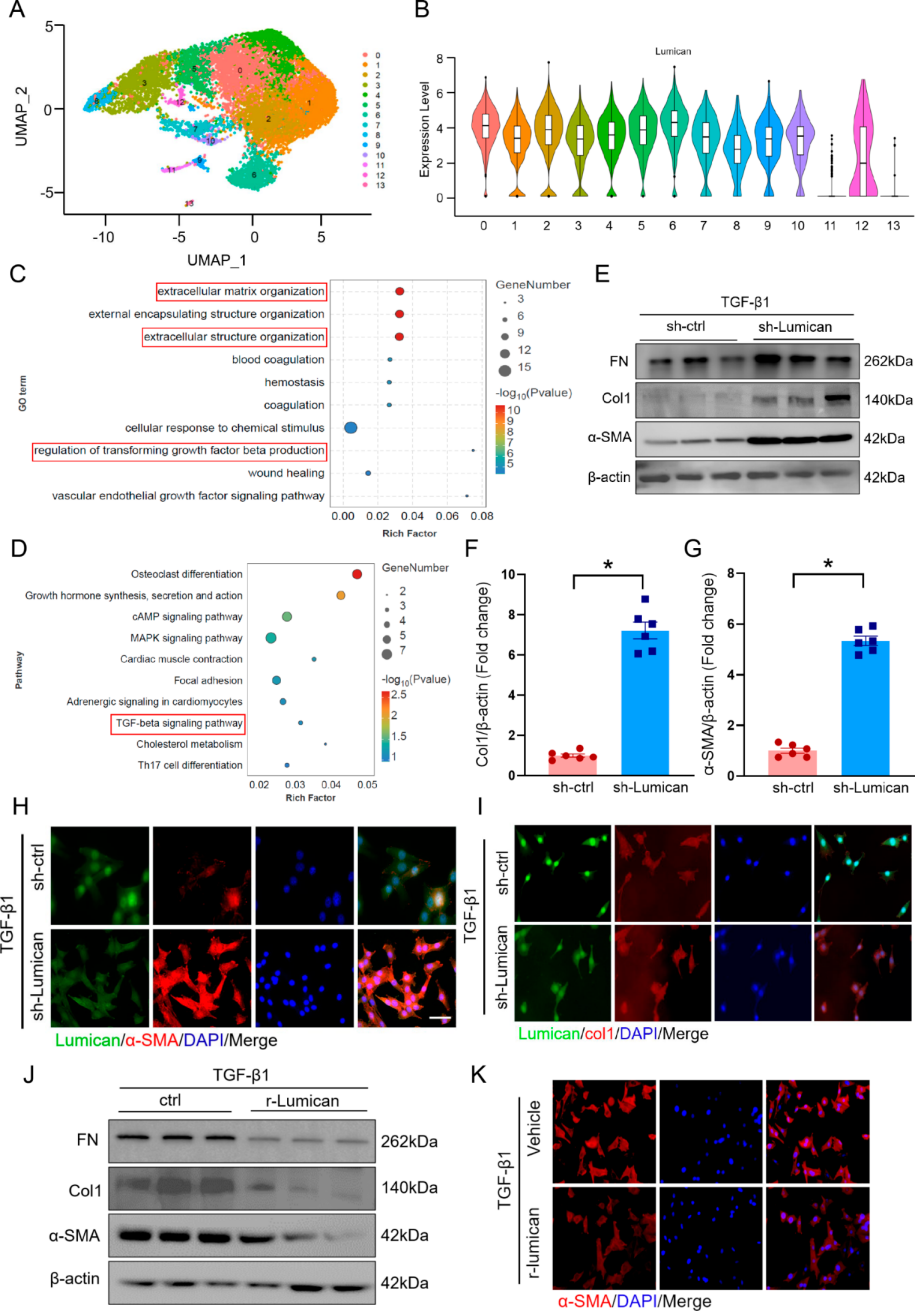
Lumican from cardiac fibroblasts regulates fibrotic phenotype conversion. **(A)** UMAP plot of fibroblasts in the infarct area tissues after MI surgery on day 14, color-coded for 13 subclusters. **(B)** Violin plots showing the normalized expression of lumican in each subcluster. **(C, D)** GO and KEGG enrichment analyses of DEGs between fibroblasts with high (Lumican^high^) and low (Lumican^low^) lumican expression. **(E)** Western blotting and quantitative analyses of α-SMA, collagen 1, and fibronectin expression in lumican-knockdown rat cardiac fibroblasts treated with 10 ng/mL TGFβ1 for 24 h. **(H, I)** Representative images of lumican (green), α-SMA (red, F), and collagen 1 (red, F) immunostaining in lumican-knockdown rat cardiac fibroblasts after TGFβ1 treatment. **(J)** α-SMA, collagen 1, and fibronectin expression in rat cardiac fibroblasts treated with 10 ng/mL TGFβ1 and 100 nM recombinant lumican for 24 h. **(K)** Representative image of α-SMA immunostaining in rat cardiac fibroblasts after treatment with TGF-β1 and recombinant lumican. The data are expressed as the means ± standard errors. Statistical analysis was performed via Student’s t test. *p < 0.05. DEG, differentially expressed gene; GO, Gene Ontology; KEGG, Kyoto Encyclopedia of Genes and Genomes; MI, myocardial infarction; UMAP, uniform manifold approximation and projection for dimension reduction

To further explore the underlying functions and mechanisms of lumican in cardiac fibroblasts during fibroblast phenotype transition, we first evaluated fibroblast subtypes in the infarcted area of MI mice. On the basis of UMAP analysis, the fibroblasts were categorized into 13 subclusters (Fig. 5A). We divided these fibroblast subtypes into lumican^high^ and lumican^low^ subclusters according to their relative lumican expression (Fig. 5B). GO enrichment analysis of DEGs between Lumican^high^ and Lumican^low^ fibroblasts revealed that genes related to fibroblast phenotype transition (FPT) were enriched (Fig. 5C). The KEGG pathway analysis confirmed these results, revealing enrichment in the TGF-β pathway (Fig. 5D). In addition, the three genes with the highest differential expression (Gsn, Htra3, and Smoc2) between Lumican^high^ and Lumican^low^ fibroblasts were associated with the TGF-β pathway (Supplementary Fig. S3B-D).

Next, we knocked down lumican in rat primary cardiac fibroblasts to characterize its role in TGFβ1-induced FPT. Lumican knockdown significantly increased the protein expression levels of α-SMA, collagen 1, and fibronectin in TGFβ1-treated cardiac fibroblasts (Fig. 5E-G, Supplementary Fig. S3A), which was supported by the results of the immunofluorescence assays (Fig. 5H-I). Furthermore, we replenished recombinant lumican in TGFβ1-treated cardiac fibroblasts. After the cultures were supplemented with exogenous recombinant lumican, the expression of these genes decreased (Fig. 5J–K, Supplementary Fig. S3). Together, these results show that lumican is involved in TGFβ-mediated FPT.

### Allopurinol ameliorates hyperuricemia-mediated adverse cardiac remodeling after MI

**Fig. 6:**
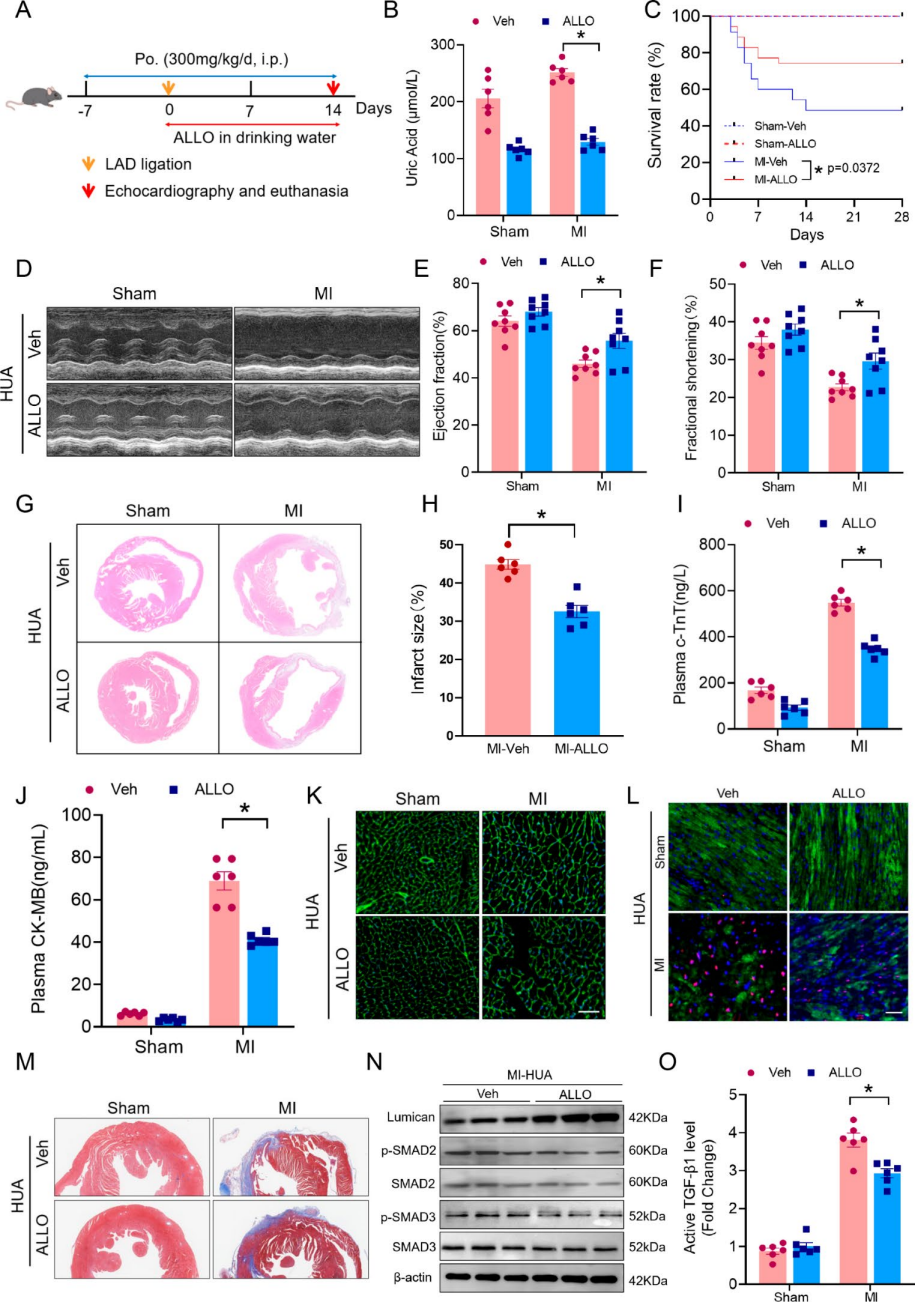
Allopurinol ameliorates hyperuricemia-mediated adverse cardiac remodeling in MI mice. **(A)** Schedule for establishing hyperuricemia and MI in a mouse model with allopurinol treatment (125 mg/L). **(B)** Serum uric acid levels in hyperuricemic mice after MI surgery, with and without allopurinol treatment, on day 14 (*n* = 6). **(C)** Kaplan–Meier survival curves for each experimental group. **(D)** Representative M-mode images of mouse hearts obtained from each group on day 14. **(E, F)** Quantification of the ejection fraction and fractional shortening measured via echocardiography on day 14 (*n* = 8). **(G, H)** Representative H&E images and quantitative analysis of cardiac tissues obtained from hyperuricemic mice on day 14 after MI or sham surgery (*n* = 7). **(I, J)** Levels of c-TnT and CK-MB in mouse plasma as assessed by ELISA (*n* = 6). **(K)** Representative images of fluorescein isothiocyanate-conjugated WGA-stained cardiac tissues from hyperuricemic mice after MI with or without allopurinol treatment on day 14. **(L)** Representative immunofluorescence images of TUNEL (red) and nuclear DAPI (blue) in c-TnT-positive cardiomyocytes (green) obtained from hyperuricemic mice after MI with or without allopurinol treatment on day 14. **(M)** Representative Masson’s staining images of cardiac tissues obtained from hyperuricemic mice after MI with or without allopurinol treatment on day 14. **(N)** Western blot analysis of lumican, p-Smad2, and p-Smad3 expression in the infarct area of hyperuricemic mice after MI surgery with or without allopurinol treatment. **(O)** Serum levels of active TGF-β1 detected by ELISA in hyperuricemia-related MI mice after allopurinol treatment (*n* = 6). The data are expressed as the means ± standard errors. Statistical analysis was performed via Student’s t test and the log-rank test. *p < 0.05. MI, myocardial infarction

We next sought to elucidate whether lowering UA levels could improve postinfarction cardiac function and repair in hyperuricemia-related MI (Fig. 6A). Compared with the control, allopurinol induced a significant decrease in UA serum levels, confirming the effectiveness of the treatment (Fig. 6B). Allopurinol treatment significantly improved post-MI survival, as 48.6% of the vehicle-treated mice survived 4 weeks after MI, which is consistent with data reported by other studies. In contrast, the survival rate of the mice treated with allopurinol increased to 74.3% (Fig. 6C). We also observed significantly greater cardiac function in the allopurinol treatment group than in the vehicle group (Fig. 6D-F). In addition, ventricular remodeling was significantly ameliorated 14 days after MI in the allopurinol-treated mice compared with the vehicle-treated mice, as illustrated by the decreased size of the infarct area as well as the decreased wall thickness (Fig. 6G-H, Supplementary Fig. S4A). Moreover, the levels of markers of myocardial injury (c-TnT and CK-MB) were decreased in the plasma after allopurinol treatment (Fig. 6I, J). Cardiomyocyte hypertrophy was also ameliorated in mice with MI after allopurinol treatment (Fig. 6K, Supplementary Fig. S4B). The number of TUNEL-positive cardiomyocytes was also significantly lower (Fig. 6L, Supplementary Fig. S4C), and the collagen density in the infarcted area 14 days after MI was significantly lower in the allopurinol-treated group than in the vehicle group (Fig. 6M, Supplementary Fig. S4D).

Allopurinol treatment reduced the p-SMAD2 and p-SMAD3 expression levels, whereas lumican expression significantly increased (Fig. 6N, Supplementary Fig. S4E-G). Also, we observed allopurinol treatment reduced the increase of active TGF-β1 in the plasma of hyperuricemia-related MI mice (Fig. 6O). These data suggest that lowering UA levels restrains hyperuricemia-mediated accelerated myocardial dysfunction and adverse cardiac remodeling after MI.

## Discussion

**Fig. 7:**
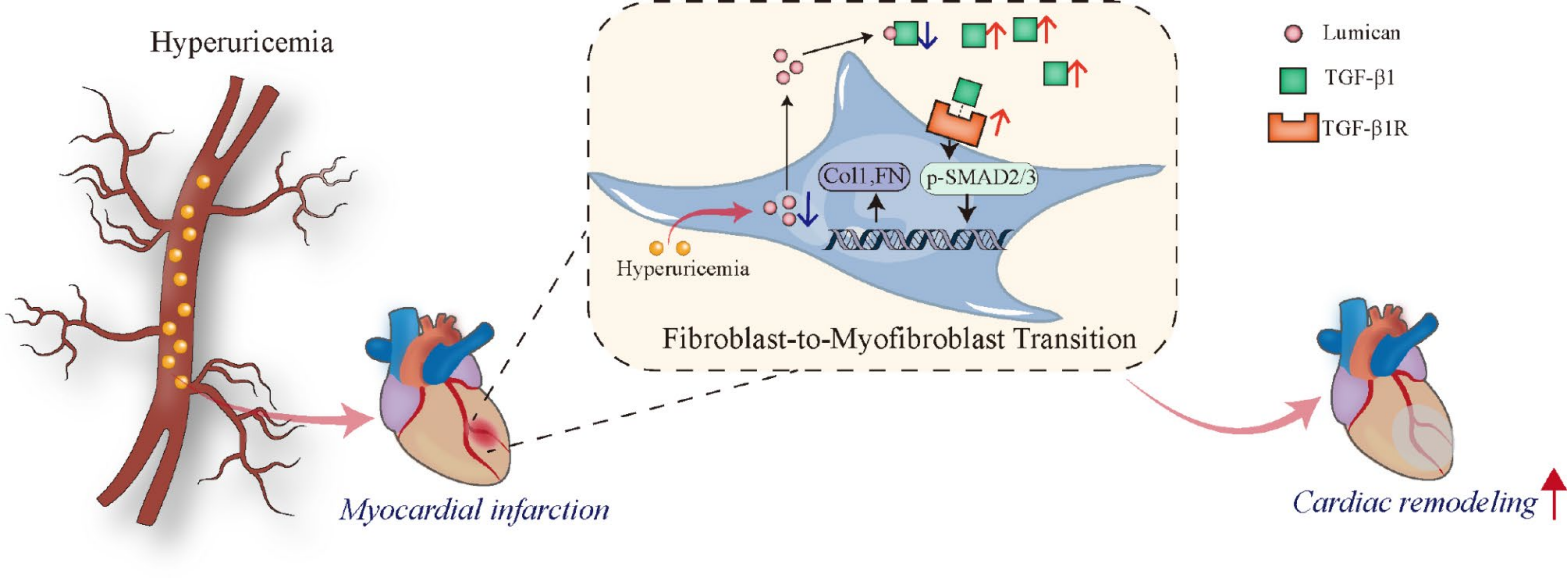
Schematic illustration of the regulatory mechanisms of the lumican/TGF-β pathway in hyperuricemia-mediated adverse cardiac remodeling

Hyperuricemia is closely associated with an increased risk of cardiovascular diseases such as heart failure, MI, and hypertension [30]. Although hyperuricemia is an independent predictor of cardiovascular mortality, the underlying mechanisms responsible for this association remain largely unknown [30, 31]. This study demonstrated that hyperuricemia plays a critical role in post-MI cardiac remodeling. Specifically, hyperuricemia impaired cardiac function, increased mortality, and aggravated adverse ventricular remodeling in experimental MI mice. Furthermore, the data confirmed that the effect of hyperuricemia is due to a shortage of fibroblast-derived lumican, which in turn promotes fibroblast activation and fibrotic function via the TGFβ/SMAD signaling pathway. Excessive ECM deposition in the infarcted area aggravates MI progression (Fig. 7).

MI is the most severe subtype of coronary artery disease and is recognized as a significant public health issue. Therefore, identifying potential risk factors and improving patient prognosis are essential [11, 32, 33]. The impact of individual lifestyle components and their interactions on cardiovascular health remains largely unexplored. Lifestyle factors significantly influence serum UA levels and associated hyperuricemia, and several population studies have identified elevated serum UA as a risk factor for coronary artery disease [34, 35]. Although the association between hyperuricemia and coronary artery disease is well known, the specific mechanisms that regulate UA remain unclear. Consequently, various mechanisms contributing to coronary artery disease have been proposed, including oxidative stress, regulation of inflammation, RAAS activation, endothelial dysfunction, UA metabolism, and cardiomyocyte injury [31, 36].

UA-lowering therapy can effectively reduce the risk of mortality associated with the disease. However, considering the diversity of comorbidities and the complexity of causative factors, we still need to develop more effective targets and preventive measures [35]. The questions that arise in the clinic are primarily concerned with treating hyperuricemia. Should treatment aim to reduce patient cardiovascular risk, and if so, at what UA level should treatment begin, and what should the target be? Here, we first demonstrated that hyperuricemia impairs cardiac function, increases mortality, and increases adverse ventricular remodeling in a mouse model. These findings suggest that hyperuricemia is a risk factor after MI, which is consistent with the findings of population studies. Reducing UA levels with allopurinol effectively abolished the negative effects of hyperuricemia on MI. Furthermore, we found that hyperuricemia decreases lumican levels secreted by cardiac fibroblasts, which underlies its negative impact on MI. These observations support the notion that lowering UA levels in hyperuricemic patients may slow MI progression and that lumican may be an effective alternative target for the clinical treatment of hyperuricemia-related MI.

Cardiac fibrosis is facilitated by the activation of resident cardiac fibroblasts that differentiate into myofibroblasts upon myocardial injury or stress [15]. Cardiac fibroblasts, which are responsible for most ECM synthesis in the cardiac milieu, constitute 10–25% of the cells that reside in the myocardium [37, 38]. Pervasive myocardial fibrosis, a hallmark of MI, is characterized by extensive collagen matrix accretion within the myocardial interstitium [39]. Myocardial fibrosis modifies the biomechanical properties of the ECM, alters the integrity of the myocardial architecture, and compromises ventricular function. These alterations are pivotal in the pathogenesis of post-MI heart failure and are associated with clinical prognosis.

Currently, effective therapeutic interventions for myocardial fibrosis are lacking. In addition, existing treatment approaches are ineffective and face challenges posed by the multifactorial etiology of MI. Therefore, elucidating the pathogenesis and related molecular cascades responsible for myofibroblast activation will enable the identification of potential therapeutic targets capable of counteracting the pathological activation of fibroblasts. Our study corroborates the role of resident fibroblasts in the remodeling processes associated with cardiac fibrosis. Furthermore, our findings indicate that hyperuricemia modulates the profibrotic activation of cardiac fibroblasts, thereby influencing the progression of MI. We posit that urate-lowering therapies may effectively attenuate deleterious cardiac fibrosis remodeling by suppressing fibroblast activation and fibrogenic activity. Consequently, we propose that hyperuricemia contributes to the complex pathophysiology of MI and propose an ancillary therapeutic modality for antifibrotic intervention.

As one of the most relevant profibrotic factors, TGFβ isoforms are upregulated and activated in myocardial pathologies, contributing to cardiac repair and remodeling by regulating the phenotype and function of cardiomyocytes, fibroblasts, and immune and vascular cells [40, 41]. Myocardial injury induces the synthesis of bioactive TGFβ, activating SMAD2/3 signal transduction and orchestrating the regulation of proteolytic enzymes, integrin expression, and specialized ECM constituents [42]. In the early stage of MI, the activated TGFβ/SMDD signaling pathway can play a protective role by promoting an ECM-preserving phenotype in fibroblasts and preventing the generation of deleterious, proinflammatory ECM fragments. However, prolonged and overactive TGFβ signaling in pressure-overloaded cardiomyocytes and fibroblasts can promote adverse cardiac fibrosis and heart dysfunction [41]. Strategically targeting TGFβ-associated signaling pathways has therapeutic potential, albeit with challenges stemming from the pleiotropic nature of TGFβ and the heterogeneity of cardiac disease states.

Consequently, developing innovative antifibrotic approaches requires validation through robust experimental models that encapsulate the heterogeneity inherent to cardiac pathophysiology [25, 43]. Lumican, a small proteoglycan found in the ECM, has been shown to interact with TGFβ, modulating fibrotic responses in cardiac, pulmonary, cutaneous, and hepatic tissues [25]. Despite previous findings on the biological impact of lumican across diverse organ systems, its function in the context of MI-associated fibrotic remodeling remains largely unknown. Our observations revealed that lumican is significantly downregulated in patients with gout and upregulated in patients with heart failure and in MI mice. However, hyperuricemia lowered lumican expression in MI mice and activated the TGFβ/Smad2/3 signaling pathway in cardiac fibroblasts to trigger adverse cardiac remodeling. Administration of either recombinant lumican or urate-lowering therapy effectively mitigated this TGFβ-driven anomalous myofibroblast activation, decelerating hyperuricemia-associated MI progression. Our investigation is the first to suggest an association between cardiac lumican concentration and MI severity in patients with hyperuricemia.

While this study provides valuable insights into the role of hyperuricemia in exacerbating MI, there are several limitations. First, our research is primarily based on a mouse model, which may limit the applicability of our findings to human cardiovascular pathology and clinical practice. Second, while we focused on fibrosis and cardiac function, the specific mechanisms by which hyperuricemia influences MI remain to be fully elucidated. Our study may not have addressed all potential pathological processes involved. Third, although we observed adverse effects of hyperuricemia on cardiac function and fibrosis, these findings have not yet been validated in clinical samples, limiting the direct translation of our results to clinical settings. Finally, our study did not account for potential interactions between hyperuricemia and other cardiovascular risk factors, which could impact the interpretation of our results. Future research should address these limitations to provide a more comprehensive understanding.

## Conclusions

Hyperuricemia dampens cardiac function, increases ECM production, promotes cardiomyocyte death, and decreases overall survival, thereby leading to MI progression. In particular, hyperuricemia contributes to MI progression by decreasing fibroblast-derived lumican levels to activate the TGFβ/Smad2/3 signaling pathway, subsequently promoting fibroblast transition to myofibroblasts and enhancing adverse cardiac remodeling. These findings support how hyperuricemia influences fibroblast activation and accelerates post-MI remodeling. Therefore, the control of serum UA levels should be emphasized in patients at risk of MI. Clinically, patients with MI should be screened for hyperuricemia and treated to prevent adverse outcomes. This study suggests that lumican is a novel therapeutic target for hyperuricemia-related MI.

## Electronic supplementary material

Below is the link to the electronic supplementary material.

Supplementary Material 1

Supplementary Material 2

## Data Availability

All data used during the current study availableare available from the corresponding author on reasonable request.

## Abbreviations

DEG: Differentially expressed gene
DMEM: Dulbecco’s modified Eagle’s medium
ECM: Extracellular matrix
EDTA: Ethylenediaminetetraacetic acid
EDV: End-diastolic volume
ELISA: Enzyme-linked immunosorbent assay
ESV: End-systolic volume
FS: Short-axis shortening
FPT: Fibroblast phenotype transition
GO: Gene ontology
KEGG: Kyoto Encyclopedia of Genes and Genomes
LAD: Left anterior descending
LVEDD: Left ventricular end-diastolic internal diameter
LVEF: Left ventricular ejection fraction
LVESD: Left ventricular end-systolic internal diameter
MI: Myocardial infarction
PCR: Polymerase chain reaction
Po: Potassium oxonate
TGF-β1: Transforming growth factor β1
TUNEL: Terminal deoxynucleotidyl transferase dUTP nick end labeling
UA: Uric acid
UMAP: Uniform manifold approximation and projection
WGA: Wheat germ agglutinin
