# Supplementary material for: Hyperuricemia suppresses lumican, exacerbating adverse remodeling after myocardial infarction by promoting fibroblast phenotype transition
Source: J Transl Med. 2024 Oct 31;22:983. doi: 10.1186/s12967-024-05778-4 (PMC11526644; doi:10.1186/s12967-024-05778-4)
Supplement: Supplementary file 1 — Supplementary Material 1 [file 12967_2024_5778_MOESM1_ESM.docx]

**Supplementary information**

**
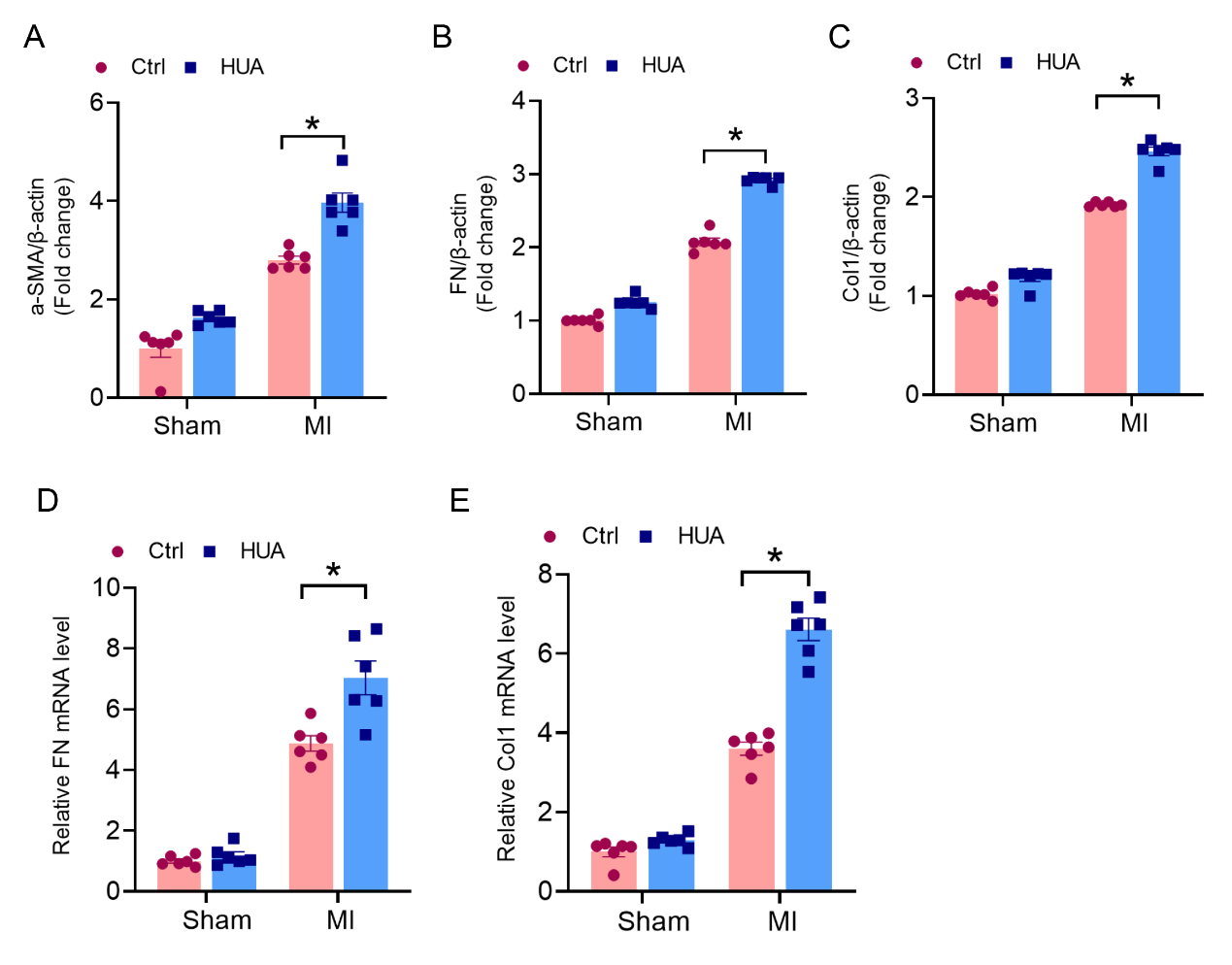
**

**Figure S1.**

A-C. Quantitative analysis of α-SMA, col 1, and FN protein expression levels in infarct area in hyperuricemic mice after MI surgery (n=6). D and E. The mRNA expression levels of Col1 and FN in infarct area in hyperuricemic mice after MI surgery.

**
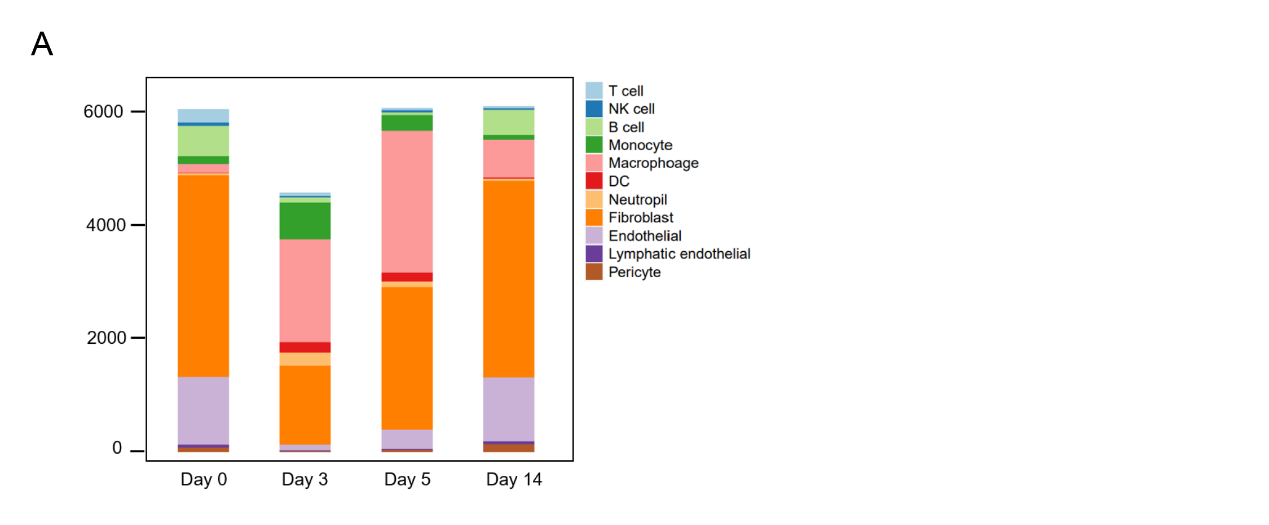
**

**Figure S2.** Number of cells of different cell types in the heart tissues after MI surgery

MI, myocardial infarction.

**
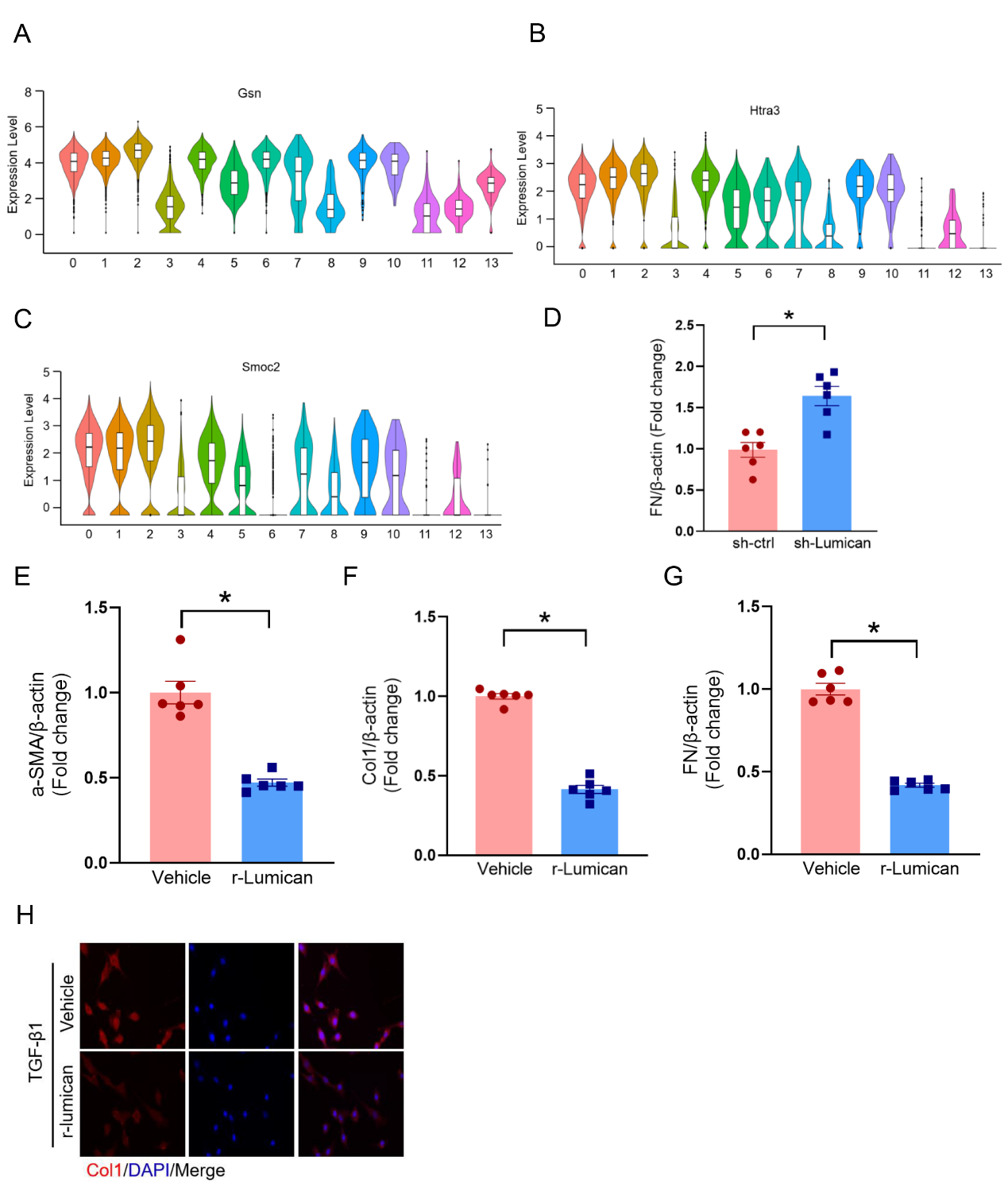
**

**Figure S3.** A. Quantitative analysis of FN protein expression levels (n=6). B-D. Violin plots show the top 3 differentially expressed genes in the lumican-high and lumican-low fibroblasts, including *Gsn*, *Htra3*, and *Smoc2*. E-G. Quantitative analysis of α-SMA, Col 1, and FN protein expression levels (n=6). H. Representative immunostaining of Col1 in rat cardiac fibroblasts after TGFβ1 and recombinant lumican treatment.

**
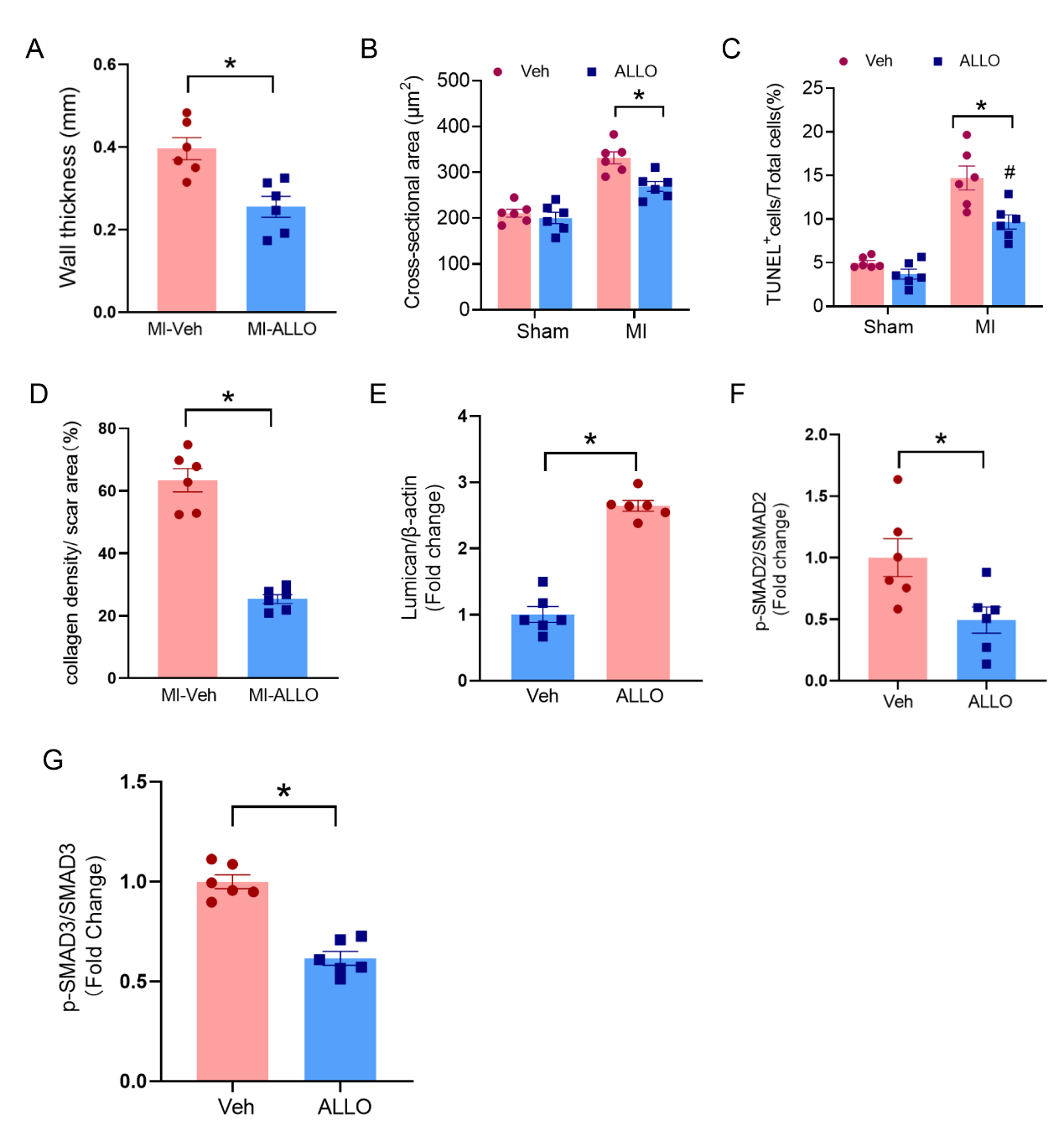
**

**Figure S4.** A. Quantitative analysis of the wall thickness after H&E staining (n=8). B. Quantificaation of cardiomyocyte cross-sectional area after WGA staining (n=6). C. Percentage of TUNEL-positive cardiomyocytes in hyperuricemic mice after treating MI with allopurinol at day 14 (n=6). D. Positive Masson’s staining area were quantified to indicate collagen density in mice heart from the indicated group (n=6). E-G. Quantitative analysis of lumican, p-Smad2 and p-Smad3 in infarct area from hyperuricemic mice after treating MI with allopurinol (n=6).

**Additional File 1**

| Primers pairs used in real-time PCR | | |
| --- | --- | --- |
| Gene name | Sense /antisense | Sequence |
| Lumican | Forward | CTCTTGCCTTGGCATTAGTCG |
|  | Reverse | GGGGGCAGTTACATTCTGGTG |
| β-actin | Forward | GGCTGTATTCCCCTCCATCG |
|  | Reverse | CCAGTTGGTAACAATGCCATGT |
| α-SMA | Forward | CCCAAAGCTAACCGGGAGAAG |
|  | Reverse | CCAGAATCCAACACGATGCC |
| Col1a1 | Forward | GCTCCTCTTAGGGGCCACT |
|  | Reverse | CCACGTCTCACCATTGGGG |
| FN | Forward | ATGTGGACCCCTCCTGATAGT |
|  | Reverse | GCCCAGTGATTTCAGCAAAGG |
