# Supplementary material for: Hyperuricemia suppresses lumican, exacerbating adverse remodeling after myocardial infarction by promoting fibroblast phenotype transition
Source: J Transl Med. 2024 Oct 31;22:983. doi: 10.1186/s12967-024-05778-4 (PMC11526644; doi:10.1186/s12967-024-05778-4)

**Full unedited gel for Figure 2E**

**Lumican**

**

**

β-actin


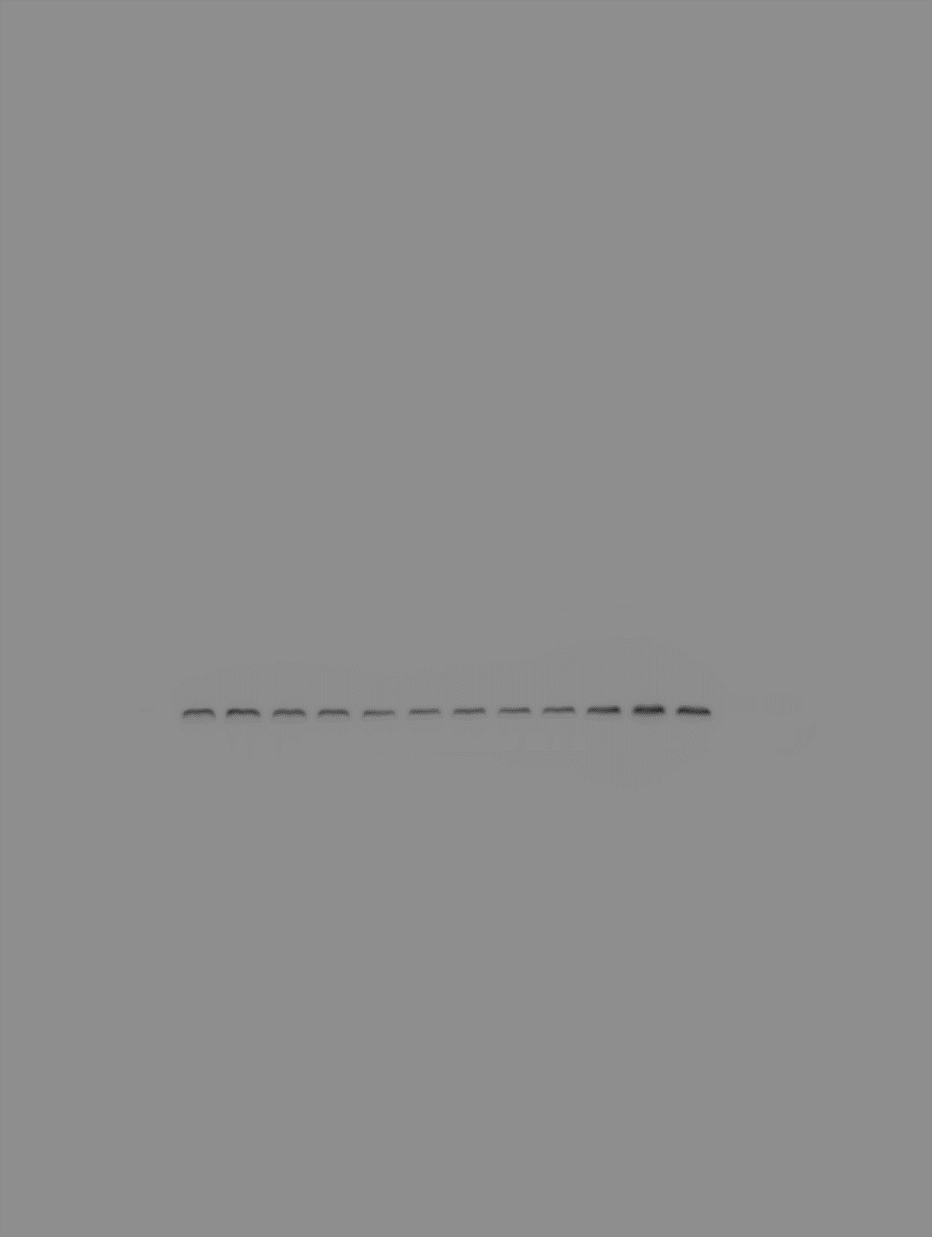


**Full unedited gel for Figure 3D**


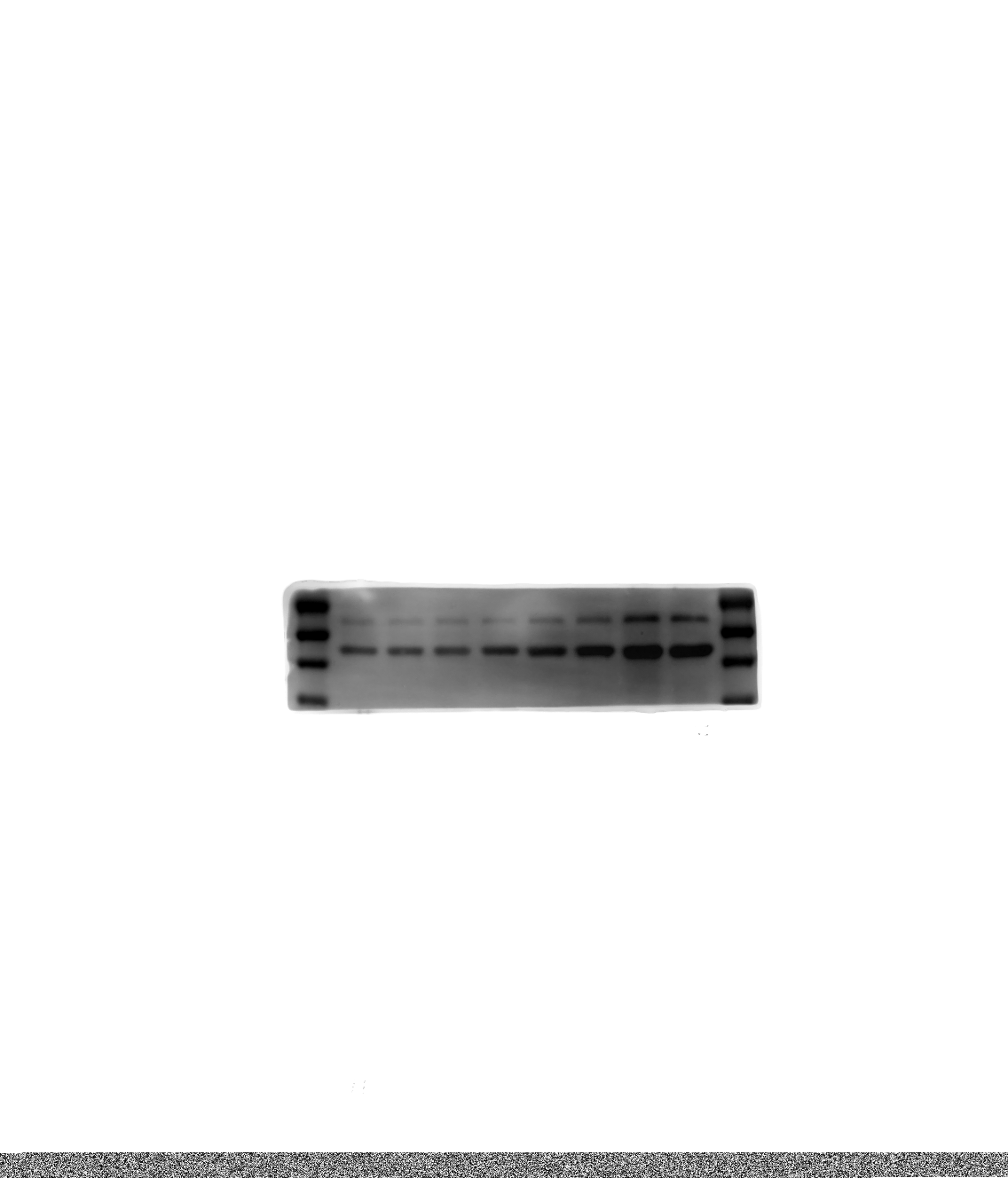
**p-SMAD2**

**SMAD2**


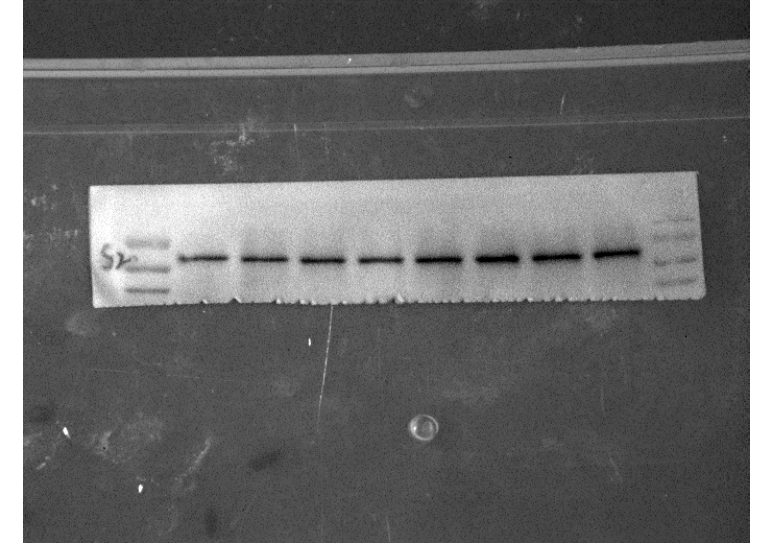


**p-SMAD3**


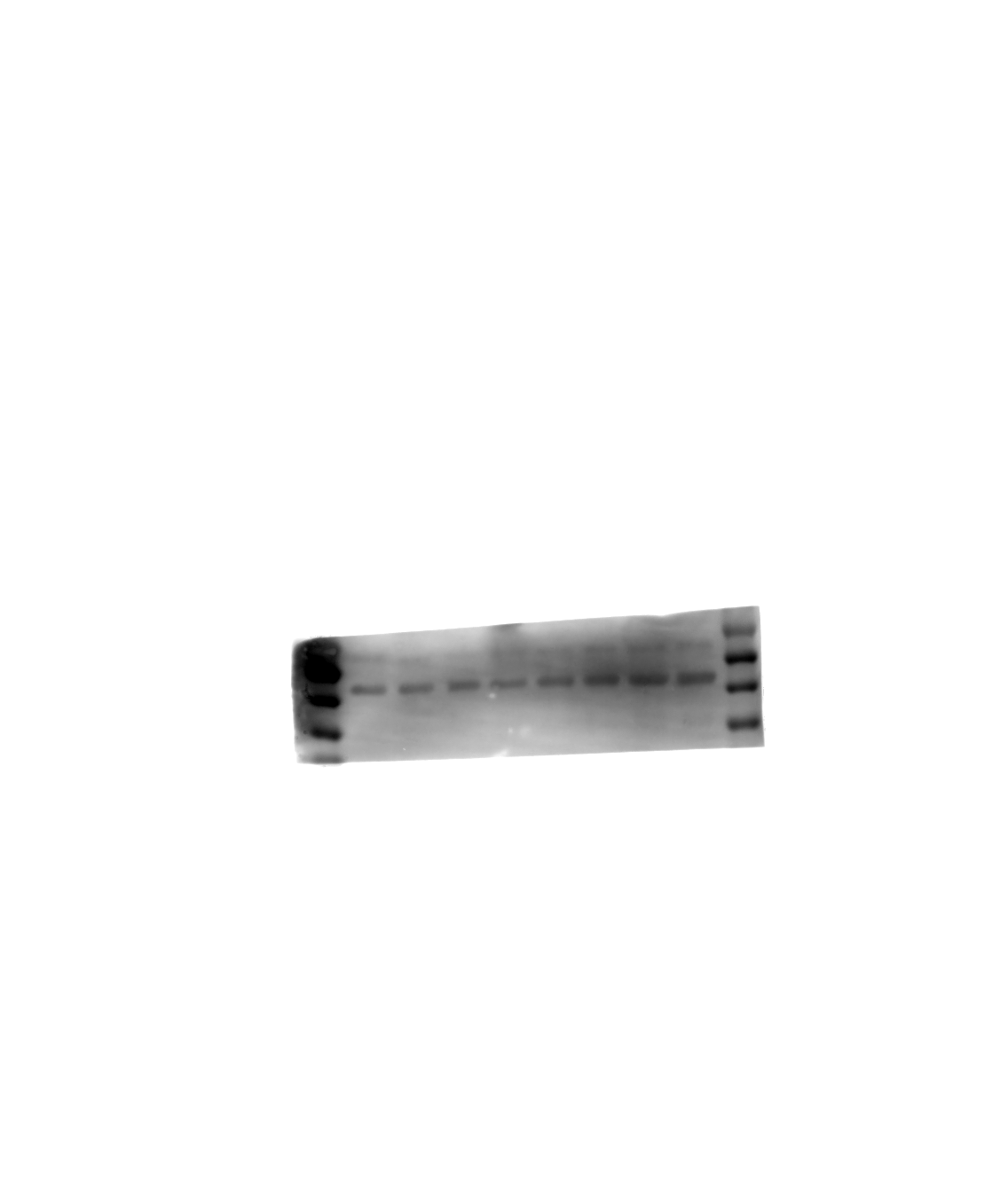


**SMAD3**


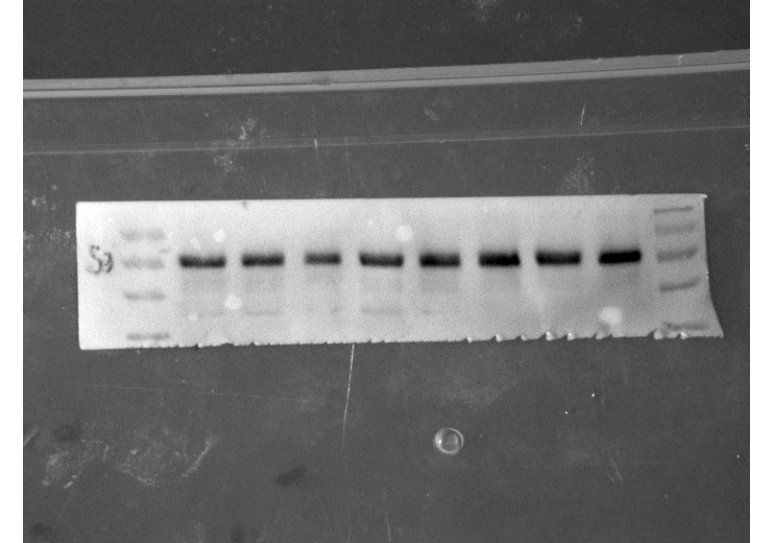

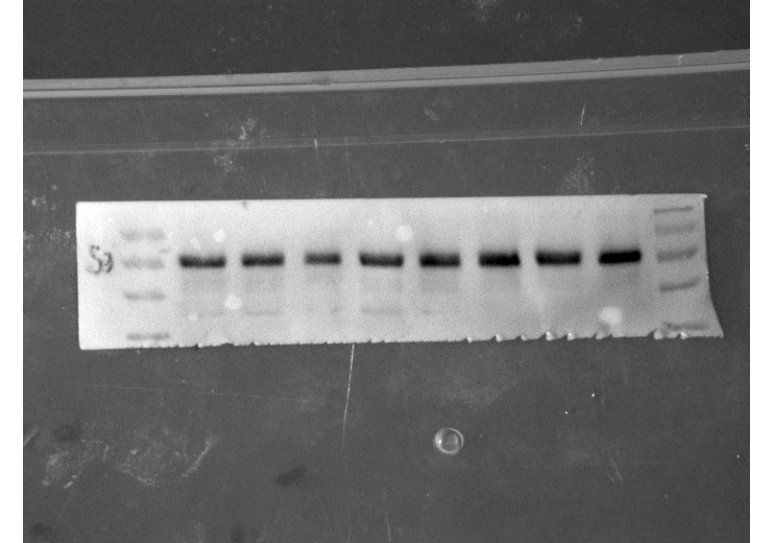


**SMAD4**


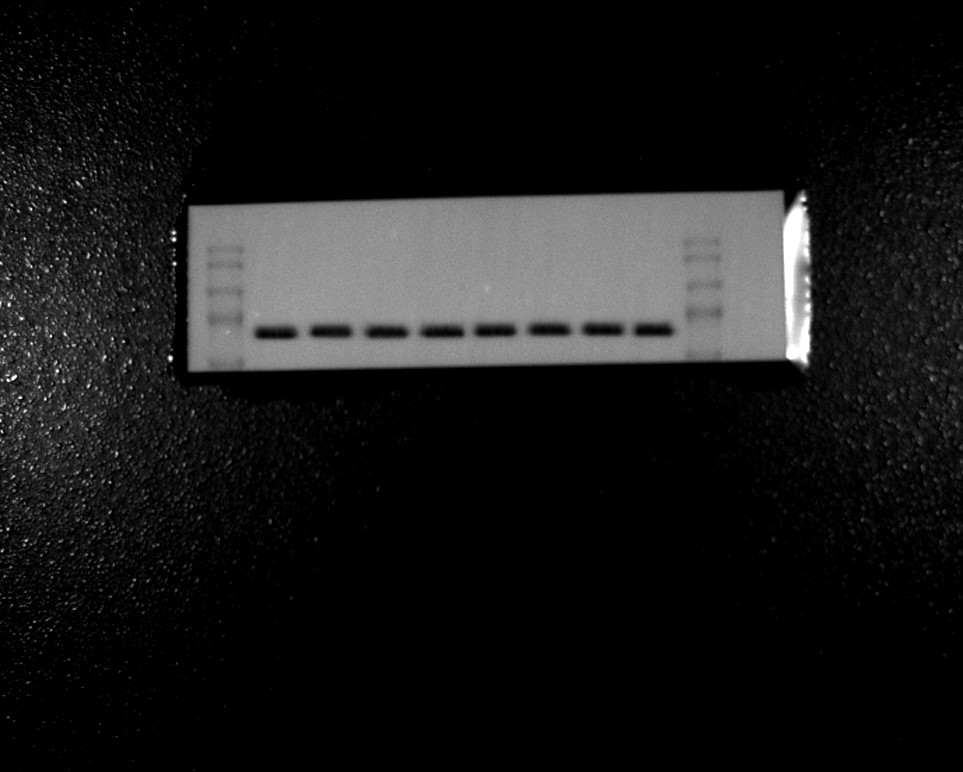


**β-actin**


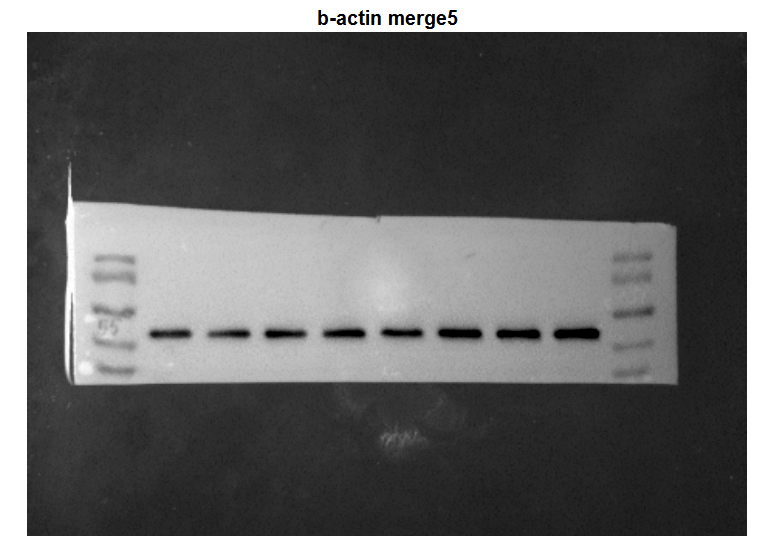


**Full unedited gel for Figure 3H**

**α-SMA**


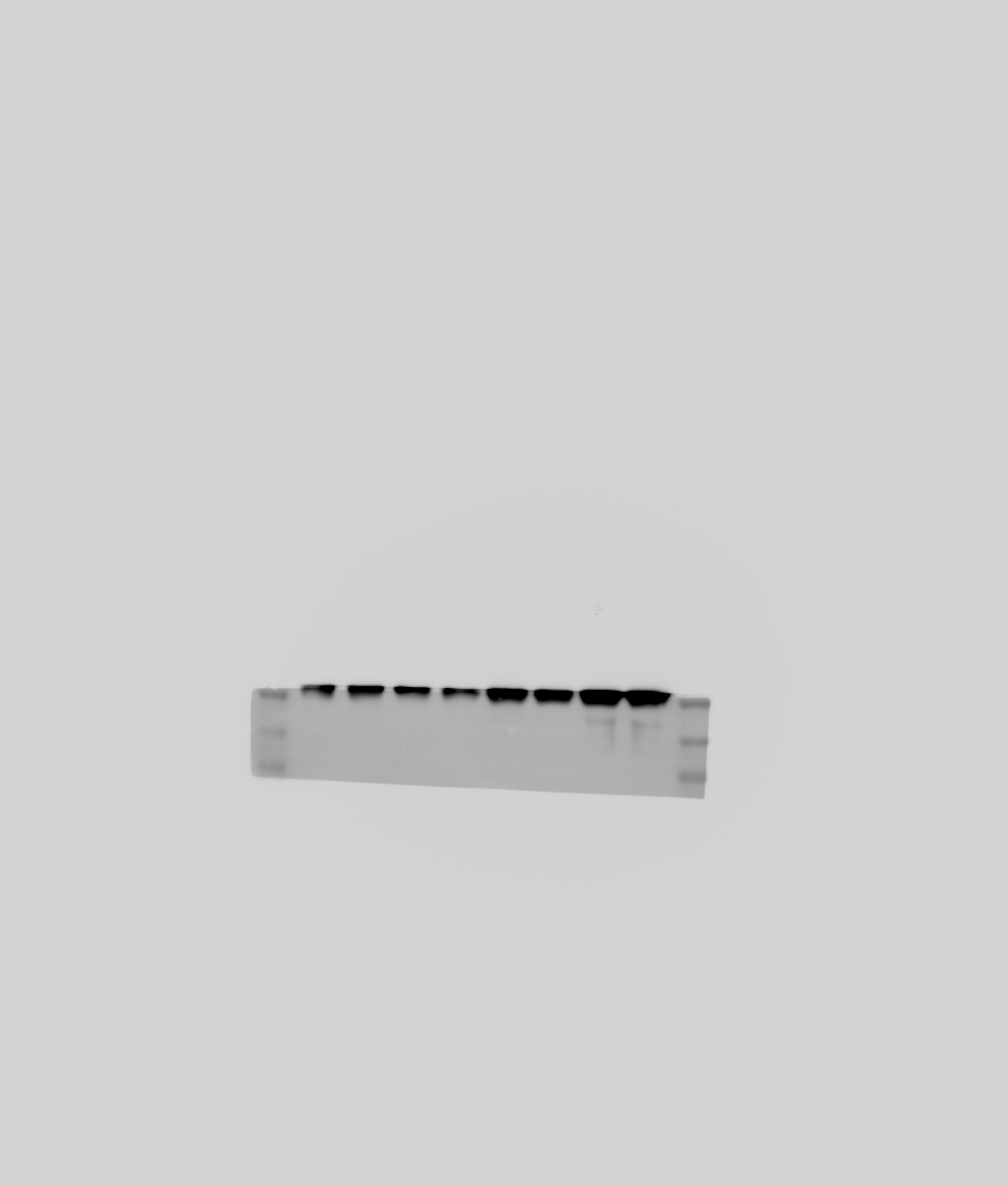


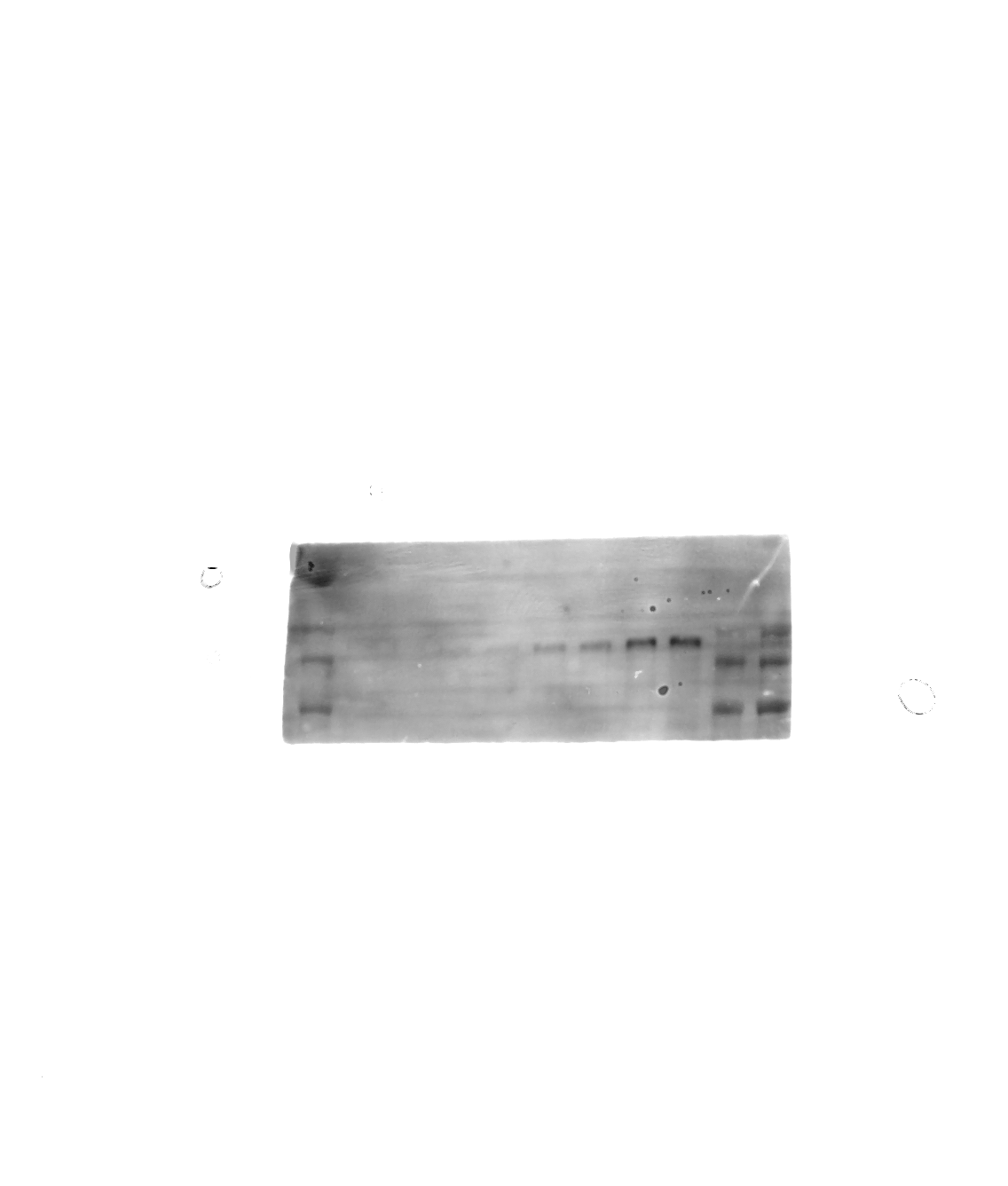
**Col1**


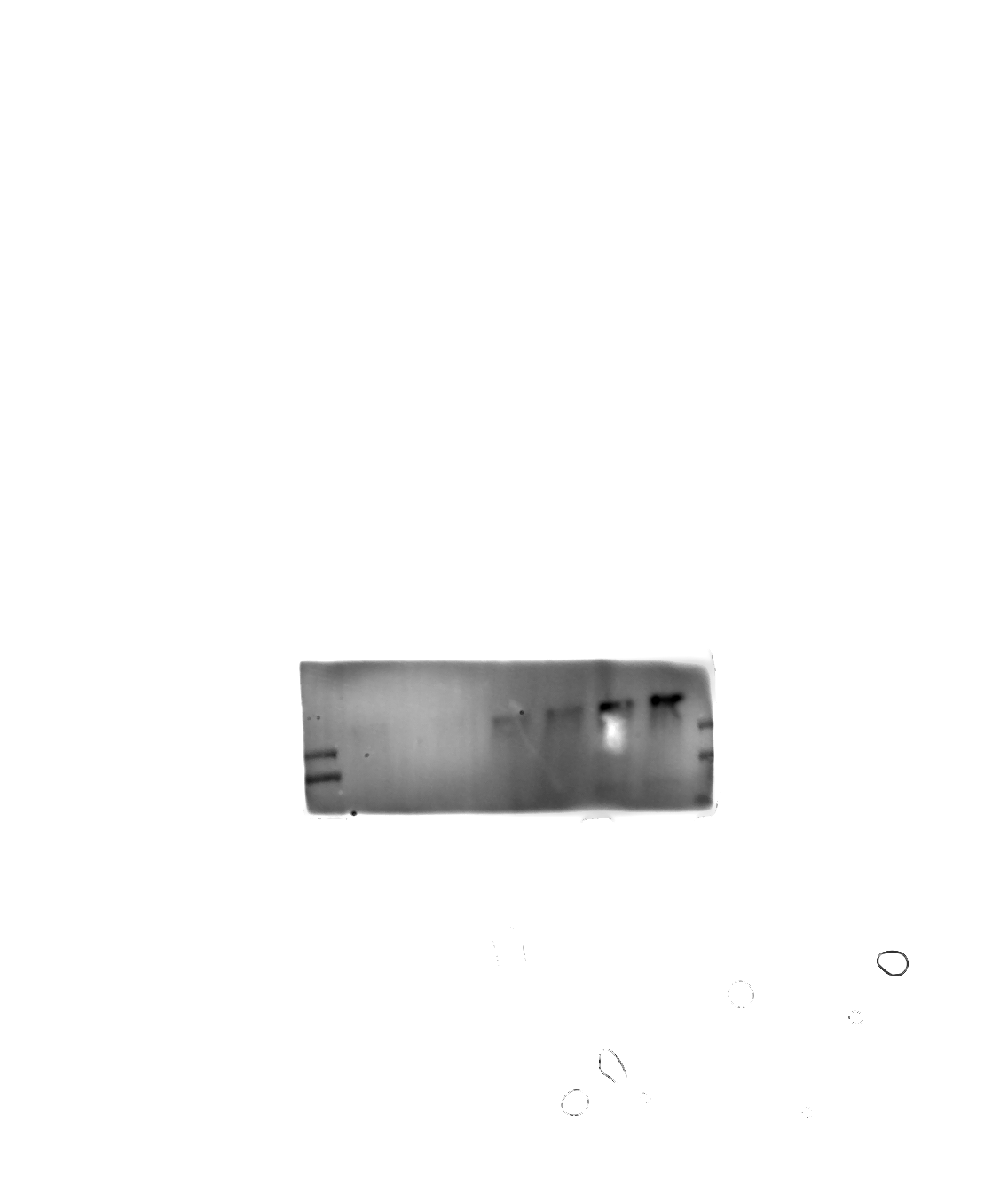
**Fn**


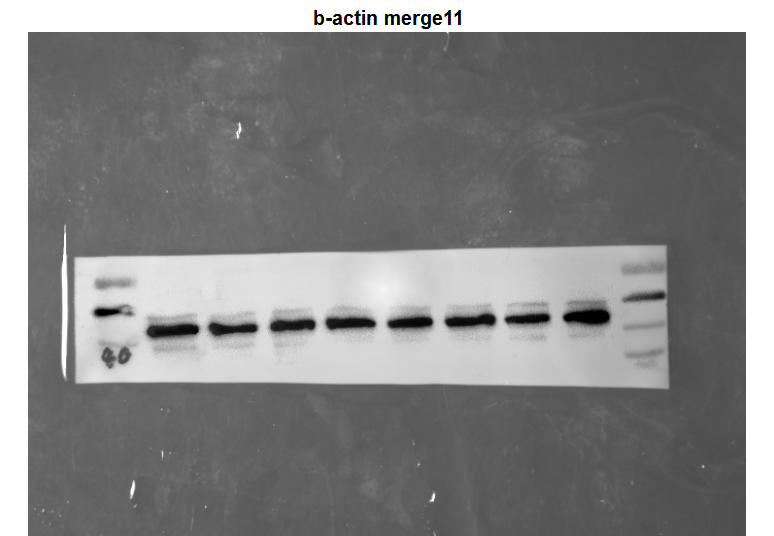
**β-actin**

**Full unedited gel for Figure.4F**

**a-SMA**


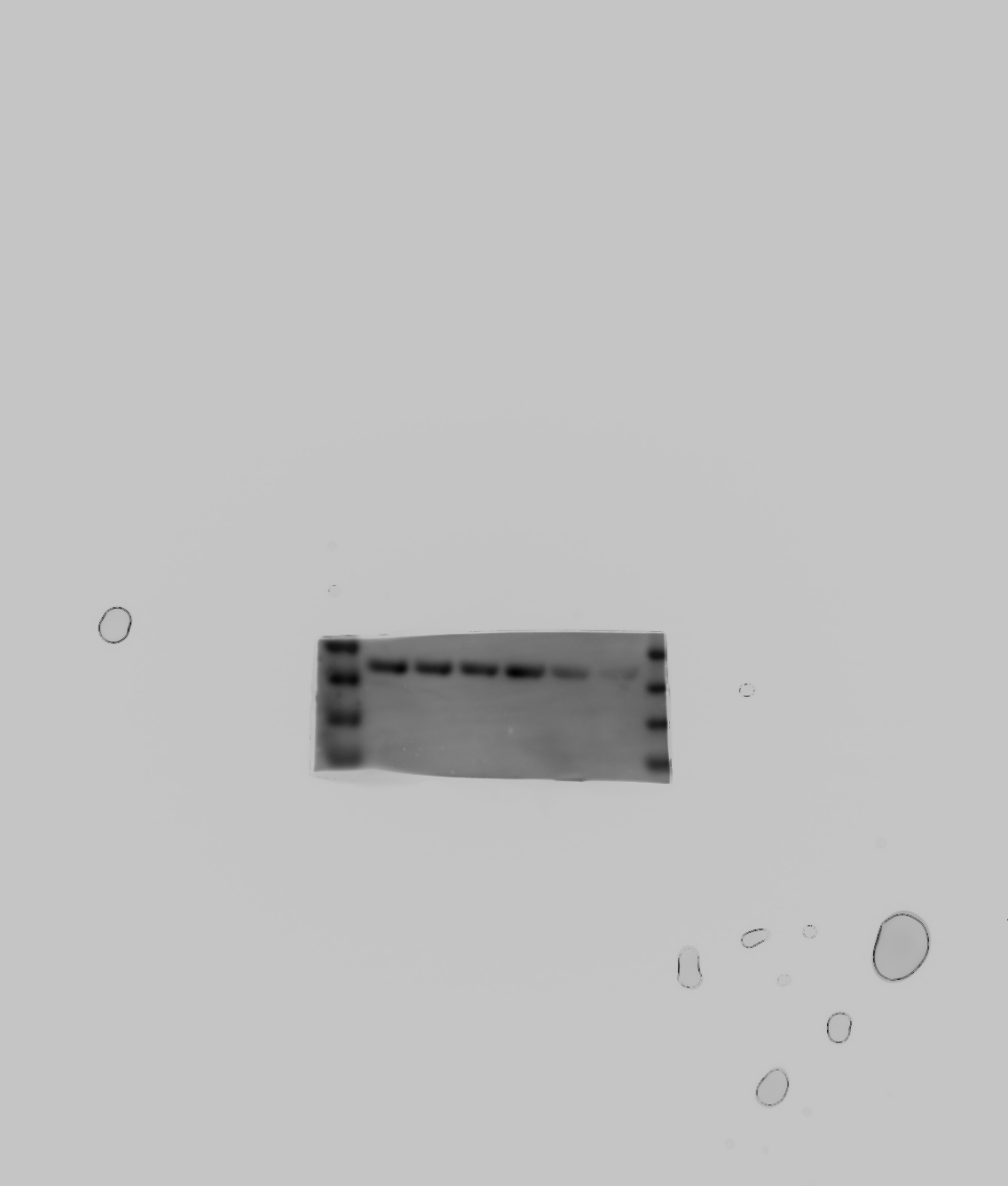


**COL1**

**
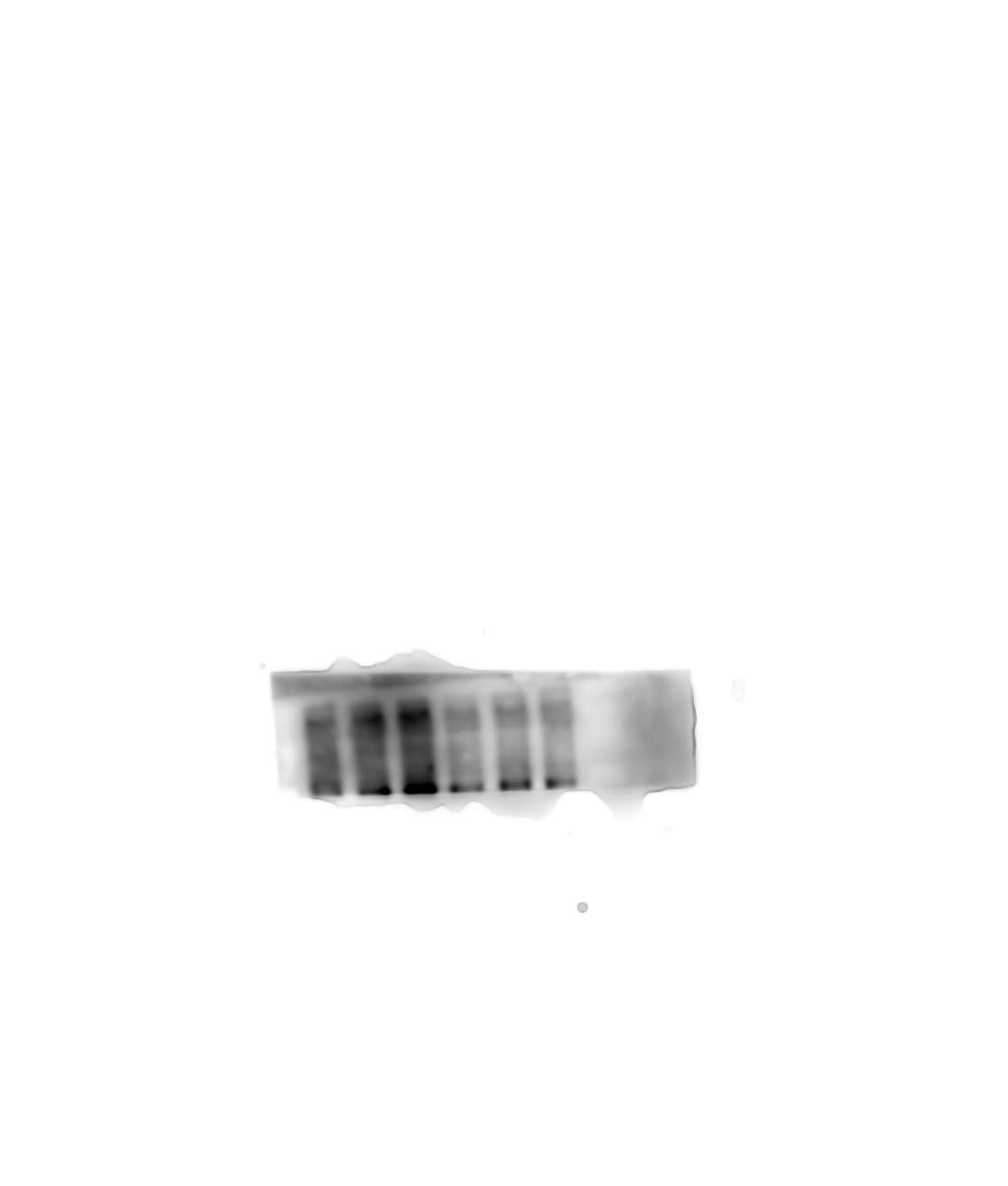
**

**Fn**


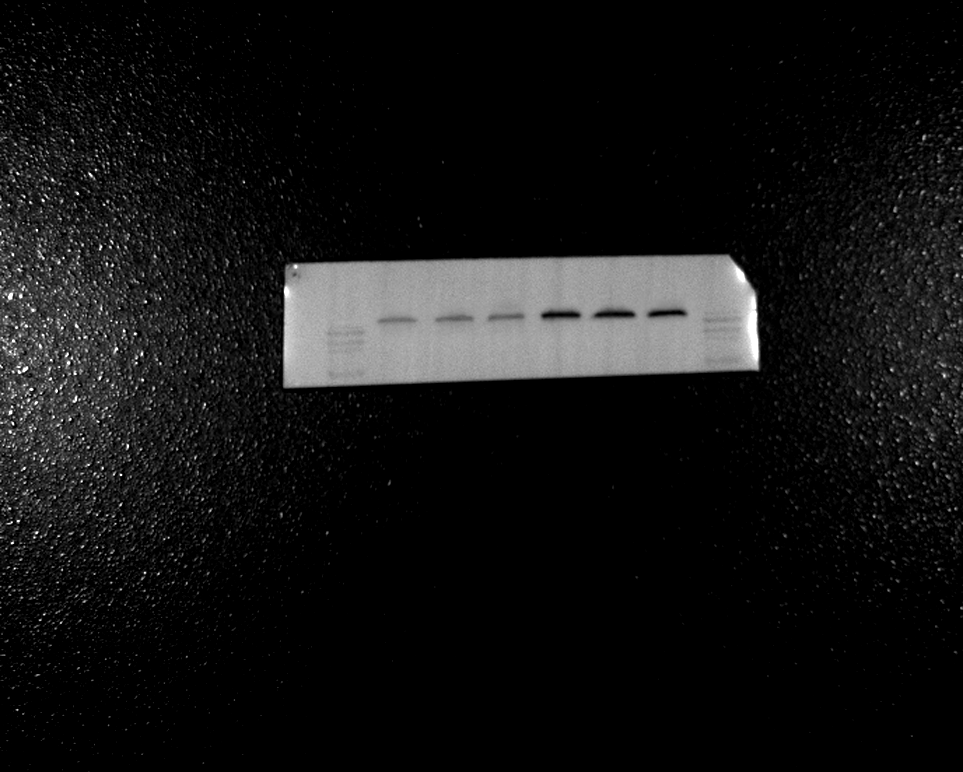


**β-actin**


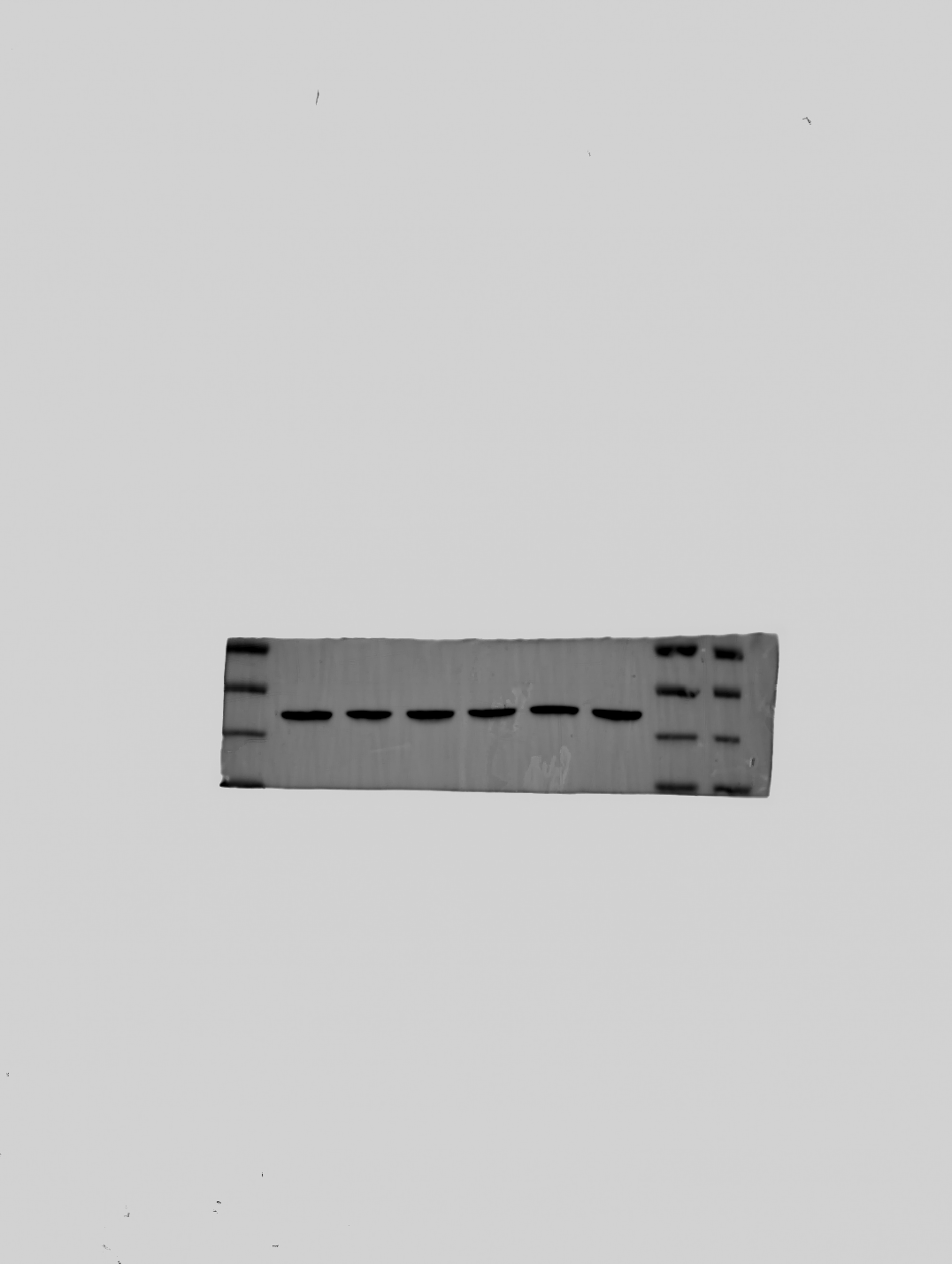


**Full unedited gel for Figure 5E**



a-SMA


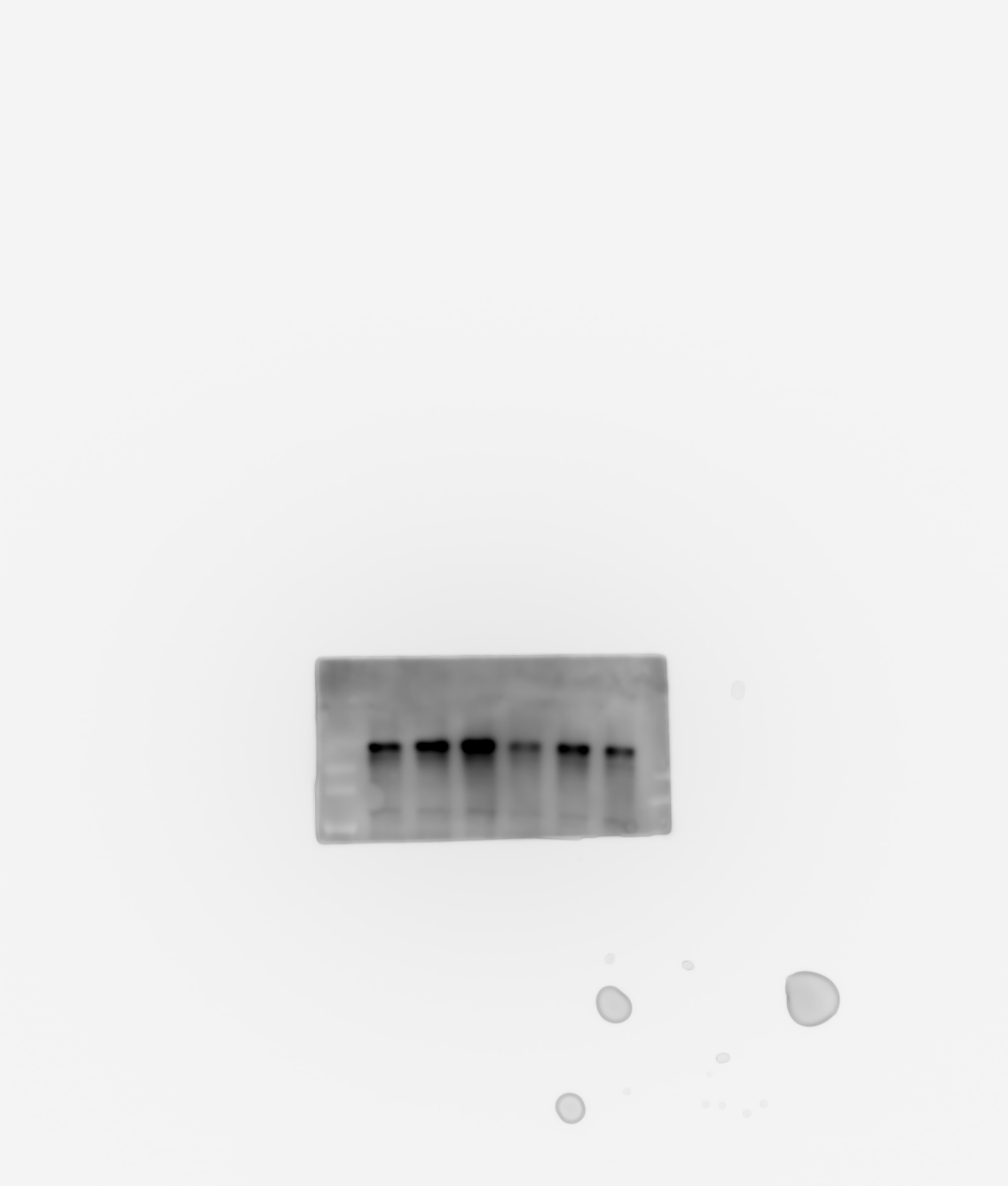


**Fn**

**COL1**


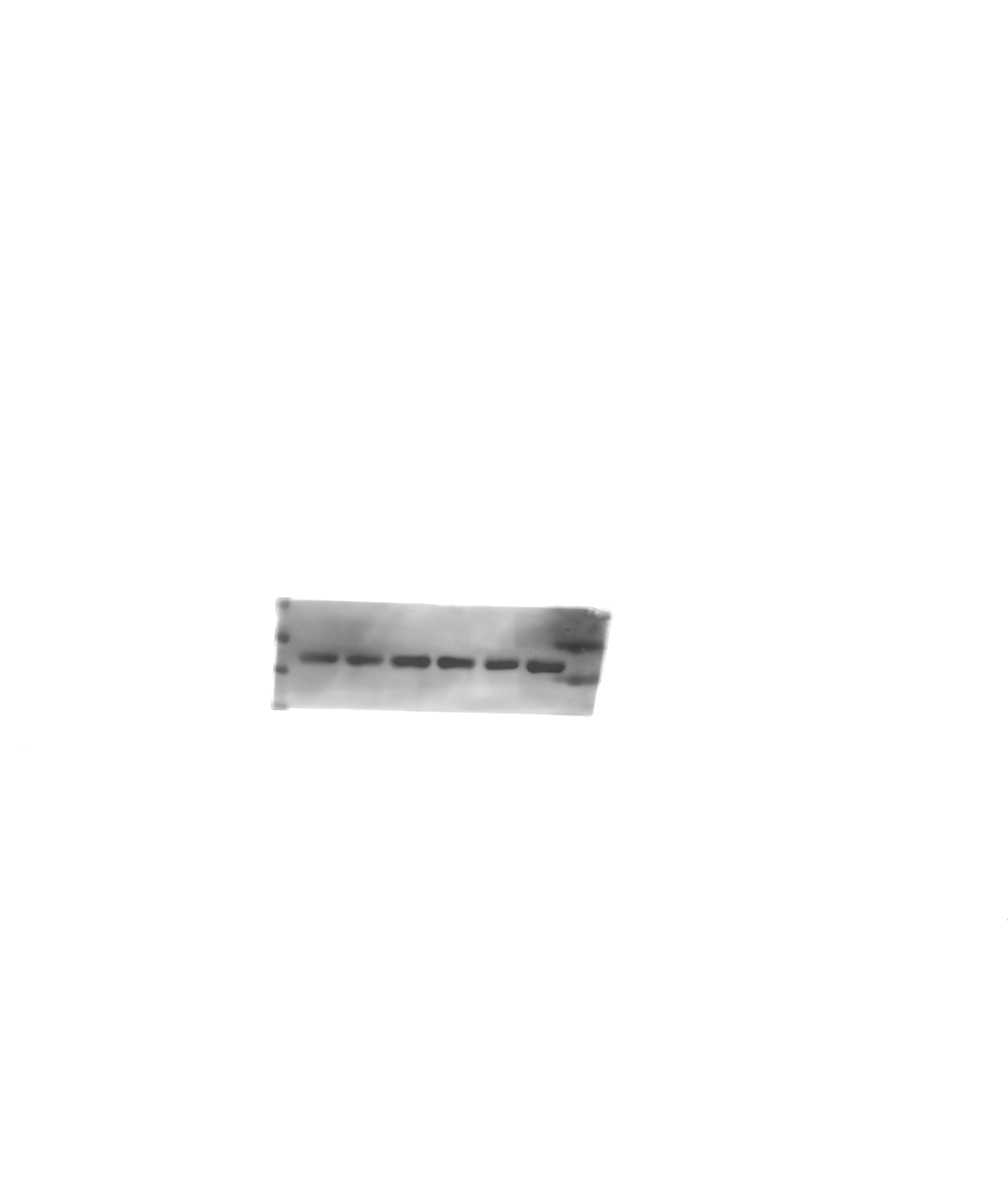
**β-Actin**

**Full unedited gel for Figure 5J**

**COL1**


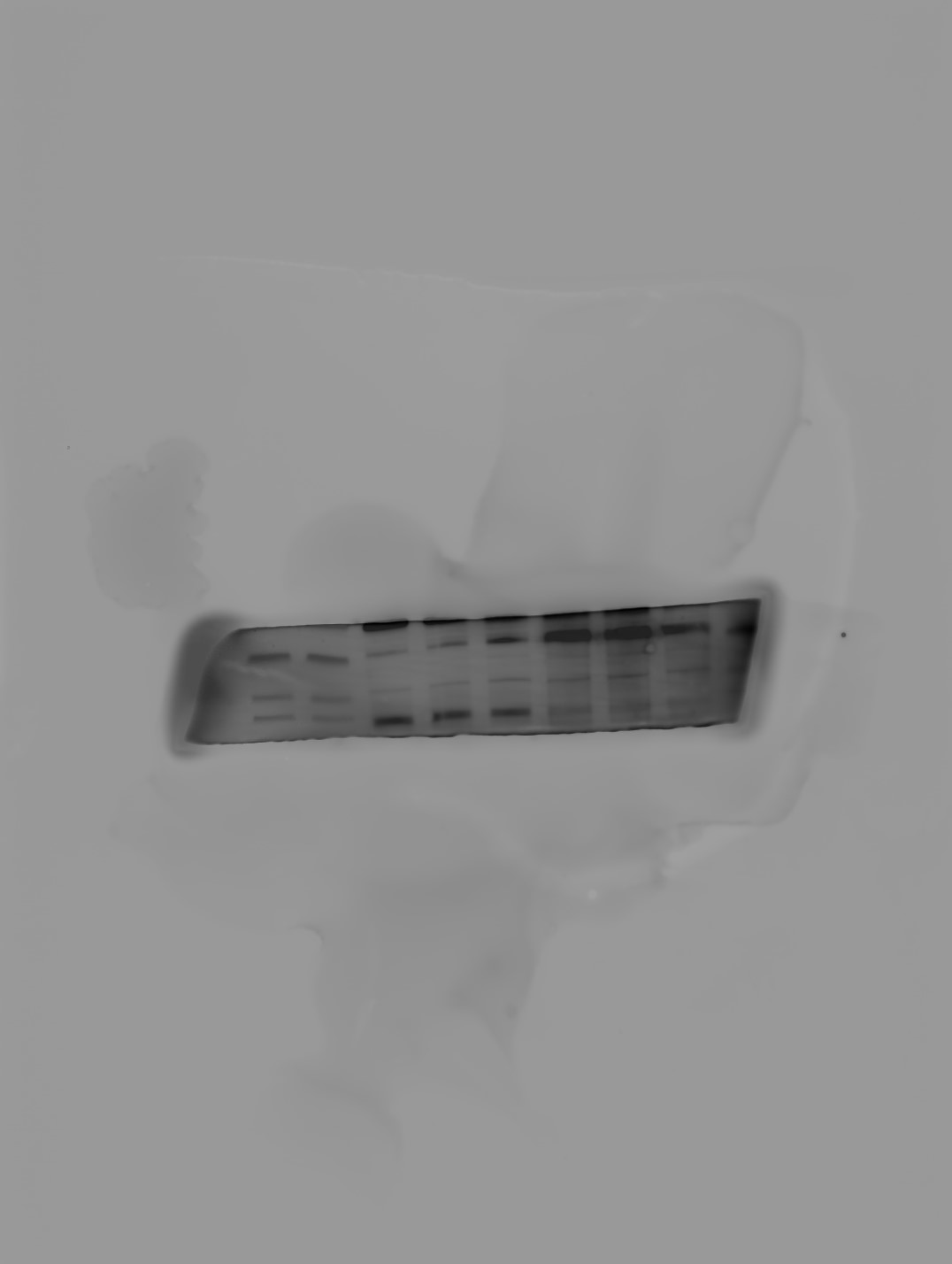


**FN**


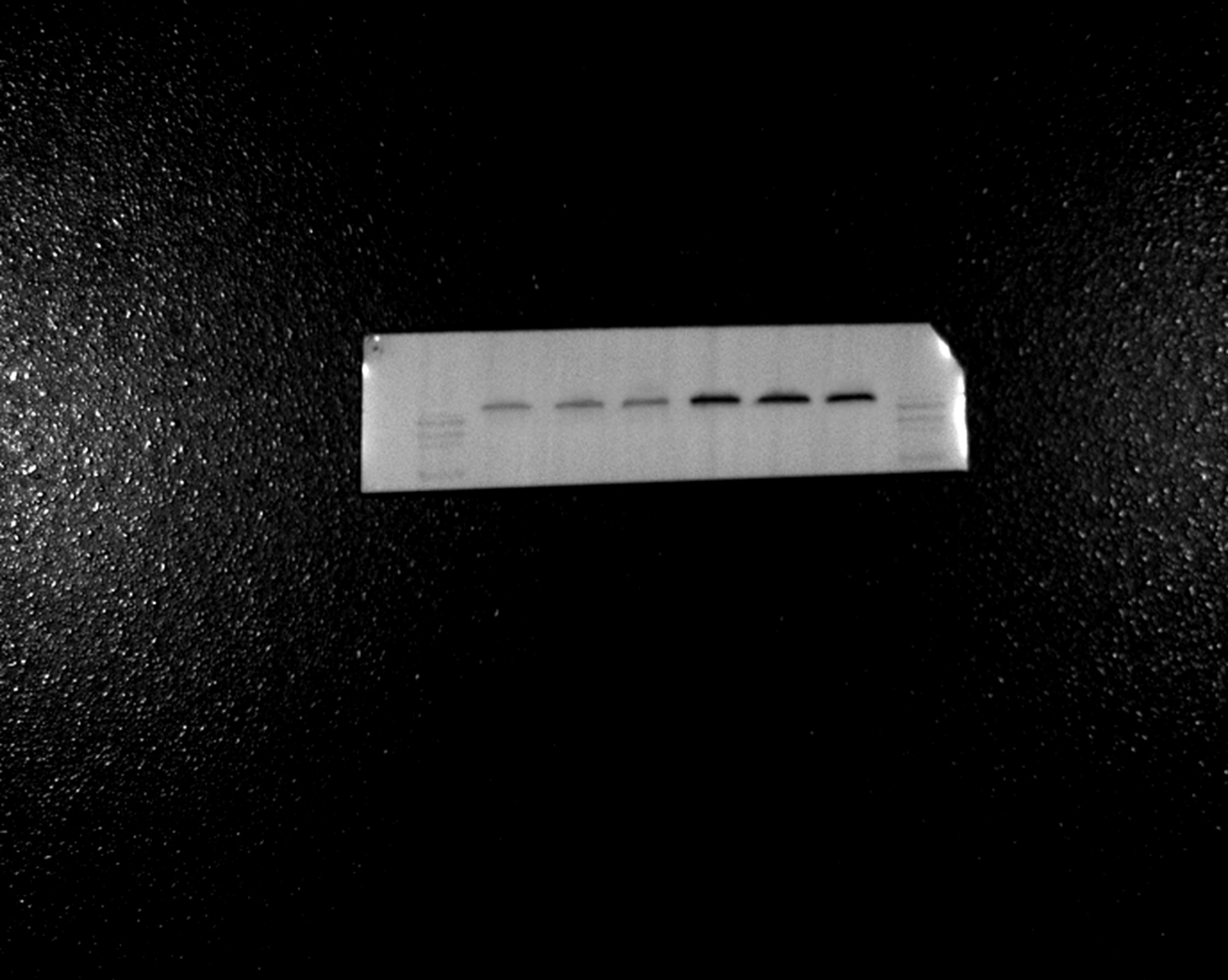


**a-SMA**


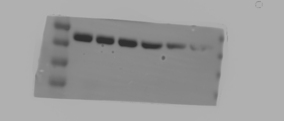


**β-actin**


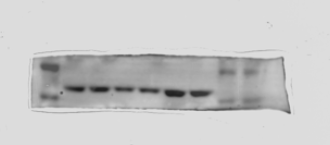


**Full unedited gel for Figure 6N**

**Lumican**


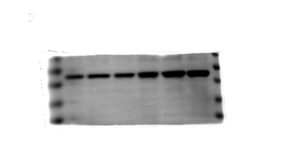


p-SMAD2


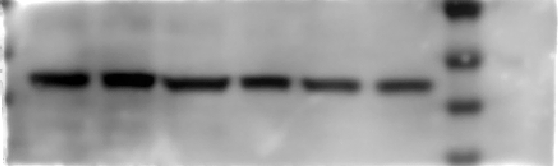


SMAD2


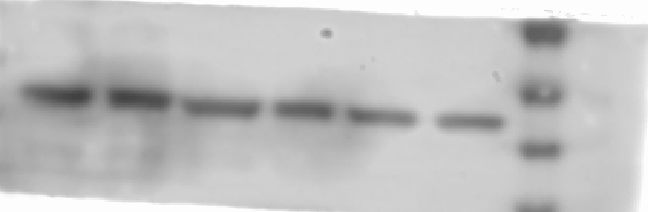


p-SMAD3


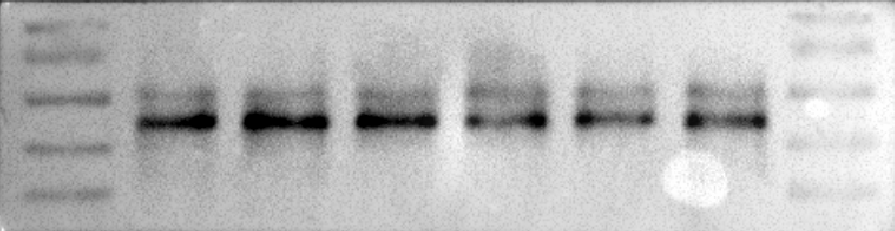


SMAD3


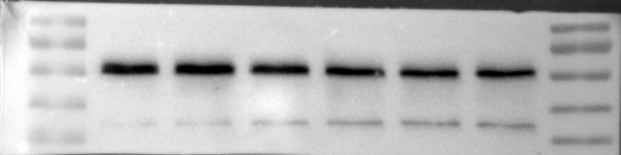


β-actin


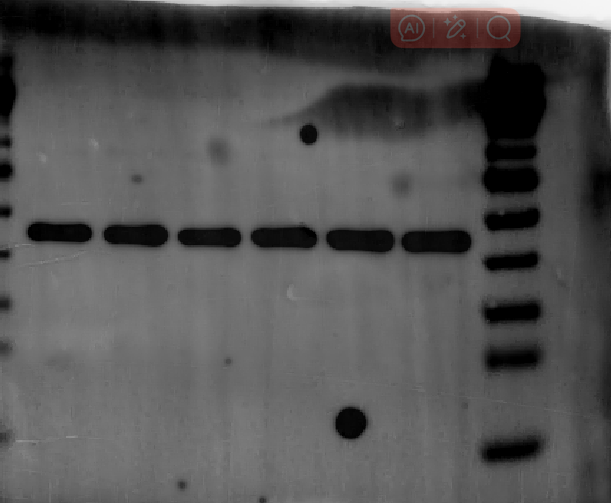

Supplement: Supplementary file 2 — Supplementary Material 2 [file 12967_2024_5778_MOESM2_ESM.docx]
